# Hyperosmotic priming of *Arabidopsis *seedlings establishes a long-term somatic memory accompanied by specific changes of the epigenome

**DOI:** 10.1186/gb-2013-14-6-r59

**Published:** 2013-06-14

**Authors:** Emanuela Sani, Pawel Herzyk, Giorgio Perrella, Vincent Colot, Anna Amtmann

**Affiliations:** 1 Institute of Molecular, Cell and Systems Biology (MCSB), College of Medical, Veterinary & Life Sciences (MVLS), University of Glasgow, Glasgow G128QQ, UK; 2 Ecole Normale Supérieure, Institut de Biologie de l'ENS (IBENS), Paris, F-75005, France; 3 Institute of Cell Biology, University of Edinburgh, Edinburgh EH9 3JR, UK

## Abstract

**Background:**

In arid and semi-arid environments, drought and soil salinity usually occur at the beginning and end of a plant's life cycle, offering a natural opportunity for the priming of young plants to enhance stress tolerance in mature plants. Chromatin marks, such as histone modifications, provide a potential molecular mechanism for priming plants to environmental stresses, but whether transient exposure of seedlings to hyperosmotic stress leads to chromatin changes that are maintained throughout vegetative growth remains unclear.

**Results:**

We have established an effective protocol for hyperosmotic priming in the model plant Arabidopsis, which includes a transient mild salt treatment of seedlings followed by an extensive period of growth in control conditions. Primed plants are identical to non-primed plants in growth and development, yet they display reduced salt uptake and enhanced drought tolerance after a second stress exposure. ChIP-seq analysis of four histone modifications revealed that the priming treatment altered the epigenomic landscape; the changes were small but they were specific for the treated tissue, varied in number and direction depending on the modification, and preferentially targeted transcription factors. Notably, priming leads to shortening and fractionation of H3K27me3 islands. This effect fades over time, but is still apparent after a ten day growth period in control conditions. Several genes with priming-induced differences in H3K27me3 showed altered transcriptional responsiveness to the second stress treatment.

**Conclusion:**

Experience of transient hyperosmotic stress by young plants is stored in a long-term somatic memory comprising differences of chromatin status, transcriptional responsiveness and whole plant physiology.

## Background

Information storage (memory) is a prerequisite for the functioning of any biological or artificial system. Where and for how long information on experienced events should be stored depends on the purpose of the memory, such as altering immediate responses, learning through repetition, or archiving for future generations. Amount of information stored and duration of storage have to be carefully managed to avoid any negative impact on speed and fitness of the system.

Memory in higher plants is evident in altered responses to environmental stimuli after pre-exposure to the same or related stimuli, termed 'priming' or 'acclimation' depending on sequence and strength of the successive stimuli. Prominent examples include seed preconditioning, temperature acclimation, and systemic acquired resistance [1-3]. Over the last 10 years research into the molecular basis of plant memory has seen a boost of activity based on emerging knowledge on the role of chromatin modifications in determining gene activity [4,5] and trait variation [6,7].

Heritability of chromatin modifications through mitosis and meiosis provides a potential mechanism for long-term storage of information on environmental events both within the life span of an individual ('somatic memory') and across generations ('trans-generational memory'). Several independent studies have reported chromatin changes (for example, loss of methylated DNA or of di-methylated lysine 9 in histone 3, H3K9me2) and re-activation of transposable elements in the offspring of environmentally challenged plants [8-12]. However, whether stress-induced chromatin changes are heritable through multiple generations and whether they underpin the acquisition of adaptive traits is still a matter of debate [13,14]. The evidence to date favors the view that stress-induced trans-generational changes of the chromatin might increase survival chances of the species, rather than each individual, by broadening the phenotypic plasticity and the genetic variation within the population [15-18].

Except for vernalization [19], the importance of chromatin modifications for long-term somatic memory of individuals is also still unclear, because in most studies carried out to date the effects of biotic or abiotic stimuli on chromatin were assessed either during or immediately after the priming treatment. Such experiments have provided evidence that environmental stimuli, such as pathogen attack [20-22] or drought [23-25], alter chromatin features, both genome-wide and at specific loci, but the consequences of these changes cannot easily be separated from other changes occurring at the same time. Given their potentially stable transmission through mitosis, and hence through growth, priming-induced chromatin marks could outlive changes in transcripts, proteins, hormones, and metabolites that will underlie more or less rapid turnover, but experimental evidence for this paradigm is sparse. In one case it was reported that a decrease of tri-methylated lysine 27 in histone 3 (H3K27me3) in the cold-responsive genes COR15A and ATGOLS3, caused by a short cold treatment of *Arabidopsis thaliana *seedlings, was still measurable 3 days after transferring the seedlings back into warm conditions although transcript levels had returned to control levels within 24 h [26]. These results have provided a first indication that H3K27me3 could be a vehicle to translate transient transcriptional changes into a long-term memory but whether the identified marks are relevant for cold-acclimation remains to be investigated.

Another problem of comparative analysis of epigenetic profiles in environmentally challenged plants arises from confounding effects of plant growth and development. For example, prolonged application of drought and salt stress alter growth and development of the plants as well as inducing senescence and cell death in older leaf tissues. The phenotypic differences between treated and un-treated plants complicate the interpretation of changes in histone modification profiles obtained from entire shoots because they will not only reflect stimulus-induced changes but also changes in the relative contributions of cell-line specific profiles to the overall organ profile [27].

In the study presented here we set out to develop an experimental protocol that allowed further investigation of the roles of histone modifications in long-term somatic memory. The prevailing concern was to apply a short and mild pre-treatment that had no apparent impact on plant growth and development, and to include a period of vegetative growth between the priming treatment and the second stress treatment that was long enough to ensure dilution and turnover of proteins and metabolites that were induced by the priming treatment. Within these constraints we had to prove that the chosen priming treatment was phenotypically effective and to test whether it caused durable changes in histone modifications. Drought and salinity were chosen as environmental stimuli because they represent a good example of a natural priming situation. Thus, in arid and semi-arid environments the vegetative growth period of plants is aligned to the wet season, and therefore exposure of plants to drought (and accompanying salinity) usually occurs early and late in plant life. However, whether exposure of young plants to drought/salinity enhances tolerance in adult plants has not been systematically tested in the laboratory. Based on the development of a controlled priming protocol we show here that a short treatment of young *A. thaliana *plants with 50 mM NaCl alters the response of adult plants to salt and drought even though both treatments are separated by an extensive period of growth in control conditions.

Four histone modifications were selected as molecular candidates for harboring a long-term somatic memory of salt stress. Di- or tri-methylated lysine 4 in histone 3 (H3K4me2 and H3K4me3) is enriched in transcriptionally active genes, and these two marks are particularly prominent among dehydration-responsive genes [23-25]. Furthermore, one of the enzymes catalyzing di- or tri-methylation of H3K4 (ATX1) has been shown to be required for transcriptional and physiological responses *A. thaliana *to dehydration [28]. H3K27me3 and H3K9me2 are mutually exclusive chromatin modifications in *A. thaliana *[29], established and maintained by Polycomb complexes and SUVH histone methyltransferases, respectively [30]. Both marks have been associated with epigenetic inheritance of gene repression, but potential roles in long-term somatic memory of abiotic stress in plants have not yet been explored. Using ChIP-Seq and ChIP-qPCR we monitored genome-wide profiles of all four modifications at high-resolution in primed and non-primed plants and identified specific alterations in the H3K27me3 profile that were maintained over a 10-day growth period in control conditions. Our study therefore provides evidence for a long-term somatic memory in plants at the physiological and at the molecular level.

## Results and discussion

### Hypersomotic priming at seedling stage alters stress responses of adult plants

**Figure 1:**
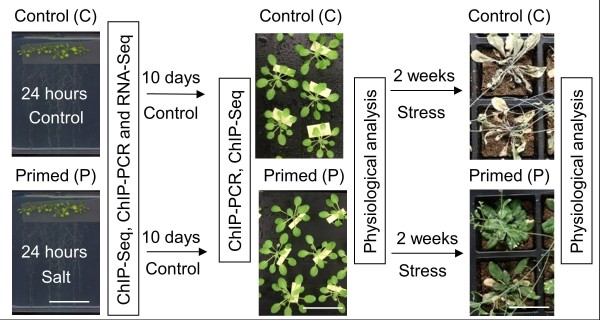
**Experimental design to investigate somatic stress memory in *A***. *thaliana. Arabidopsis thaliana *plants were germinated on vertical agar plates. Growth medium supplemented with NaCl (or not, control) was applied directly to the roots (priming) of 3-week-old seedlings. After 24 h seedlings were transferred to hydroponics or to soil and grown for another 10 days without salt. A second treatment was then applied either by adding NaCl to the hydroponic solution (salt stress) or by withholding water from soil-grown plants. Epigenetic, transcriptional, and physiological differences between primed and non-primed plants were analyzed at the indicated times. Size bars in the photos: 4 cm.

**Figure 2:**
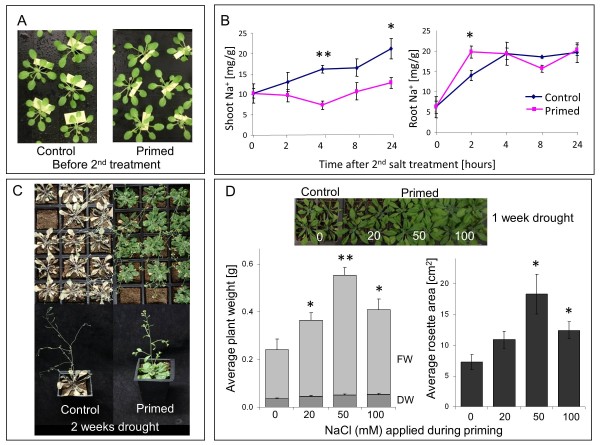
**Salt priming at seedling stage alters responses of adult plants to salt and drought**. (**A**) Appearance of primed and non-primed (control) plants after 10 days of growth in control conditions. Plants had been subjected to a 24-h treatment with 0 (control) or 50 mM NaCl (primed) at four-leaf seedling stage on agar plates, and subsequently transferred to hydroponics. (**B**) Shoot and root Na content in primed (pink) and non-primed (control, blue) plants after addition of 80 mM NaCl to the hydroponic solution of plants that had grown for 10 days in control conditions after priming. Means ± SE of four individual plants are shown. Significant differences between primed and non-primed plants are indicated with * for *P *<0.05 and ** for *P *<0.01. (**C**) Appearance of primed and non-primed (control) plants 2 weeks after onset of drought stress. (**D**) Weight and seize of plants one week after onset of drought stress. Plants subjected to different concentrations of NaCl (0 to 100 mM) during the priming treatment were analyzed. Dry weight (DW) is the horizontally dashed portion of fresh weight (FW) bars (vertically dashed). Each bar is the mean of 6-10 plants ± SE. Significant differences between primed and non-primed plants are indicated with * for *P *<0.05 and ** for *P *<0.01. Asterisks apply to both FW and DW.

Based on a series of preliminary studies altering timing, strength, and duration of pre-treatments we developed an effective protocol for hyperosmotic priming of *Arabidopsis thaliana *plants including the following steps (Figure 1): Four-leaf stage seedlings grown on vertical agar plates received a 'priming treatment' consisting in the direct application of nutrient solution supplemented with 50 mM NaCl (priming) or not (control) to the roots. Twenty-four hours later all seedlings were transferred to soil or to hydroponics, and grown in control conditions for 10 days. During this period of time the plants increased their fresh weight by eight-fold in hydroponics and 15-fold in soil. A 'stress treatment' was then applied to both primed and non-primed plants either by withholding water from soil-grown plants (drought stress) or by adding 80 mM NaCl to hydroponically grown plants (salt stress). Primed and non-primed plants were identical in size and appearance over the 10-day growth period (Figure 2A) and they did not differ in their sensitivity towards long-term (10 days) salt stress in hydroponics (Additional file 1, Figure S1). Nevertheless, over the first 24 h after salt application, primed plants accumulated significantly less Na in their shoots than non-primed plants (Figure 2B). Primed plants also showed a markedly higher tolerance to drought stress. Two weeks after onset of drought, non-primed plants displayed strong symptoms of desiccation while primed plants were still green (Figure 2C). Biometric assessment of plants at an earlier stage of drought exposure revealed a dose-dependent effect of the priming treatment on plant growth under water-limited conditions (Figure 2D). One week after onset of drought, all plants were still green but weights and rosette diameters were larger in plants primed with 20, 50, or 100 mM NaCl than in non-primed plants. Best growth in water-limited conditions was achieved when 50 mM NaCl was used for priming, and this concentration was used for subsequent molecular analyses.

The results prove that plants have indeed a mechanism for long-term storage of information about transient exposure to a mild salt treatment at seedling stage, which allows them to respond better to a second stress exposure. The observed priming effects were in accordance with the notion that during the short priming treatment the seedlings experienced osmotic stress and increased Na-influx but did not experience Na-toxicity, which is the main stress factor during long-term exposure to high salt. Importantly, early exposure to a 24-h mild salt treatment did not alter plant growth or development in unstressed conditions, and therefore any effects on subsequent stress responses must have been caused by molecular processes that did not impact on overall plant performance. Furthermore, any hormones, proteins, and metabolites generated in response to the priming treatment could be expected to underlie turnover and dilution during the growth period that separated the priming event from the second stress event. Chromatin marks were therefore the prime candidates for carrying the established memory.

### A mild hyperosmotic priming treatment alters histone modification profiles in seedlings

To investigate whether the short, mild priming treatment had an effect on the epigenome, chromatin was isolated from the roots of primed and non-primed plants and immuno-precipitated with antibodies against histone modifications H3K4me2, H3K4me3, H3K9me2, and H3K27me3. Plants were harvested immediately after the 24-h priming (or control) treatment in three independent experiments each using approximately 300 plants per condition. Enrichment of histone modifications in previously identified regions of the genome was confirmed for each sample by qPCR ('quality control', Additional file 1, Figure S2) before each set of replicate samples was combined and sequenced by Illumina technology. Reads were counted over 200 bp windows to obtain genome-wide profiles ('landscapes') of the four modifications. Files containing read counts from all samples are provided as additional files 3 to 14 (see Methods) in a format that can be readily uploaded into the Integrated Genome Browser IGB [31]. At low resolution, the obtained histone modification landscapes reproduced the basic properties of previously published profiles, such as absence (H3K4me2, H3K4me3, H3K27me3) and presence (H3K9me2) of specific modifications around centromeres [32-34], and showed no obvious differences between primed and non-primed samples (Additional file 1, Figure S3). However, further analysis of the profiles at high resolution revealed several important differences between primed and non-primed samples, which will be described in the following.

**Figure 3:**
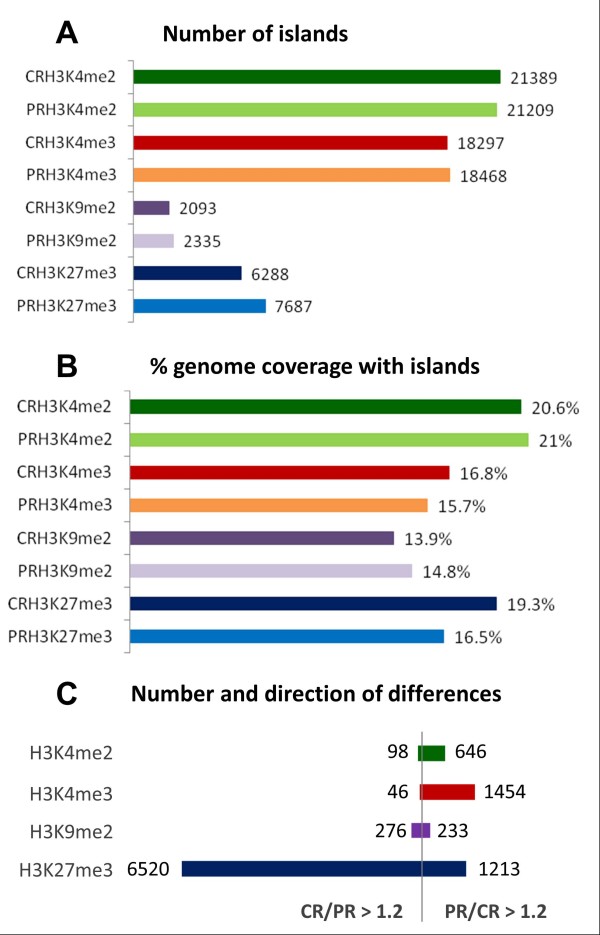
**Effect of priming on genome-wide histone modification profiles**. (**A**) Total number of continuous stretches of DNA ('islands') associated with specific histone modifications in roots of primed (PR; light colors) and non-primed (CR; dark colors) plants as determined by SICER [35]. (**B**) Total coverage of the genome with islands of specific histone modifications (in percent of whole genome sequence length). (**C**) Numbers of differences in genome-wide histone modification profiles identified by CHIPDIFF [36]. Numbers of differential sites that showed an increase of read count in the primed sample over the non-primed sample (PR/CR >1.2) are plotted to the right those that showed a decrease (CR/PR >1.2) are plotted to the left of the vertical bar. Data were obtained from pooled root material representing three independently treated plant batches of approximately 300 plants each.

Enriched histone modification domains ('islands') were determined with SICER software [35]. The highest number of islands (around 20,000) was identified for H3K4me2 and H3K4me3, followed by H3K27me3 (around 7,000) and H3K9me2 (around 2,000). Lists specifying the positions of identified islands in all samples are provided as Additional files 17 to 28 for upload into IGB (see Methods). The number of H3K4me2, H3K4me3, and H3K9me2 islands was similar in primed and non-primed plants, but the number of H3K27me3 islands increased from 6,288 in non-primed to 7,687 in primed plants (Figure 3A). The overall genome coverage with H3K4me2, H3K4me3, and H3K9me2 islands was again similar in primed and non-primed plants but genome coverage with H3K27me3 islands decreased from 19.3% in non-primed plants to 16.5% in primed plants despite the higher number of islands (Figure 3B).

### Priming induces small but specific changes in histone modification levels

CHIPDIFF software [36] was used to identify genomic regions that differed in histone modification level between primed and non-primed samples. CHIPDIFF extracts only those sites that display differences between the two samples that are significantly larger than those in the neighboring regions thereby taking into account the overall noise in the profiles. Positions of all identified differences between primed and non-primed root samples are provided as additional files for upload into IGB (Additional files 31 to 36, see Methods). The number of identified differential sites depended on the lysine residue under consideration. H3K27me3 showed the largest number of differences, followed by H3K4me3 and H3K4me2, while H3K9me2 produced the least differences (Table 1). In most cases, the relative changes in the identified sites were small; only H3K27me3 displayed differences larger than two-fold. To investigate whether small differences in the H3K4me2 and H3K4me3 profiles could nevertheless be biologically meaningful we compared differences in the root samples to those in separately analyzed shoot samples of the same plants (primed root/non-primed roots and primed shoots/ non-primed shoots). Using a cutoff of 1.2-fold, we identified only 12 (H3K4me2) and 20 (H3K4me3) differences in shoots compared to 744 (H3K4me2) and 1,500 (H3K4me3) differences in roots (Table 1). Considering that roots but not shoots were in direct contact with the priming solution the finding provided strong support for a causal link between the priming treatment and the identified differences. At a higher cutoff (1.5-fold) no differences were found in the shoot samples. The fact that high-resolution shoot profiles from primed and non-primed plants were virtually identical indicated that the pooling of 3 × 300 plants had eliminated treatment-independent variation within and between the replicate batches of plants.

**Table 1:** Number of differences^a ^between primed and non-primed samples

Differences in roots (*n*)	H3K4me2	H3K4me3	H3K9me2	H3K27me3
>2-fold	0	0	0	1678
>1.5-fold	71	105	10	3912
>1.2-fold	744	1500	509	7733

Differences in shoots (*n*)	H3K4me2	H3K4me3	H3K9me2	H3K27me3

>1.2 fold	12	20	nd^b^	nd^b^

^a^Identified by CHIPDIFF.

^b^Not determined.

The direction of priming-induced changes in methylation also depended on the specific lysine residue (Figure 3C). Only for H3K9me2 the number of differential sites that showed an increase in read count in primed plants was similar to the number of sites showing a decrease. For H3K4me2 and H3K4me3, the vast majority of identified differential sites showed a higher read count in the primed than in the non-primed sample, and many of the identified sites showed an increase in both di- and tri-methylation of H3K4. By contrast, the vast majority of differential H3K27me3 sites showed a lower read count in the primed than in the non-primed sample. Opposite effects on H3K4 and H3K27 methylation consistently favor a more open chromatin structure in primed plants.

**Figure 4:**
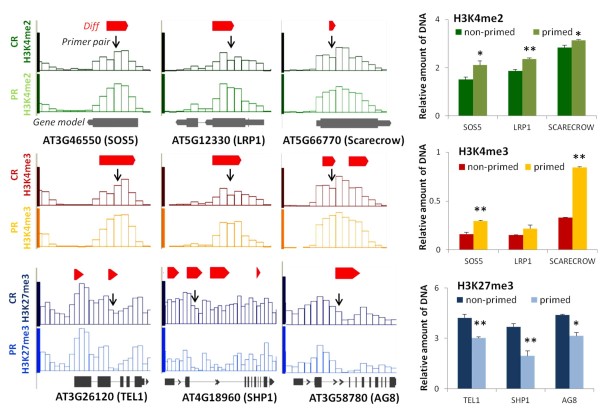
**Confirmation of individual histone methylation marks in primed roots**. Position and verification of differential sites for H3K4me2 (green), H3K4me3 (red/yellow), and H3K27me3 (blue). Differences identified by CHIPDIFF are indicated with red bars above the ChIP-Seq profiles on the left (displayed in IGB). Profiles of non-primed roots (CR) are in shown in dark colors, those of primed roots (PR) in light colors. Black arrows indicate genome positions of the fragments amplified by qPCR. Average relative amounts of DNA amplified by qPCR for the indicated sites are shown in the bar graphs on the right. Each value was normalized against input and reference. References were constitutively di- or tri-methylated regions in At2g24560 (for H3K4) or At5g56920 (for H3K27). Bars are means ± SE of three independently treated replicate plant batches each consisting of approximately 300 plants (same material as pooled for ChIP-sequencing). Significant differences between primed and non-primed plants are indicated with * for *P *<0.05 and ** for *P *<0.01.

On the basis of the ChIP-Seq profiles we designed primer pairs within identified differential sites, and carried out qPCR using the ChIP samples from the individual replicate experiments. Dual normalization against input DNA and a constitutive reference region allowed direct quantitative comparison of the PCR amplifications. Figure 4 shows that qPCR faithfully reproduced the site-specific small differences of H3K4me2, H3K4me3, and H3K27me3 that had been identified by CHIPDIFF in the genome-wide profiles. Priming-induced changes in H3K9me2 were not further analyzed in this study.

In summary, we found that the priming treatment did not lead to major remodeling of genome-wide histone modification profiles, but introduced small changes on top of the well-established landscapes of the four histone modifications tested. This could be expected since chromatin structure is essential for defining tissue identity and guiding developmental programs, and these fundamental functions should be robust against fluctuations in the environment. Indeed, the fact that we compared morphologically identical plants after a short, transient environmental stimulus sets our study apart from previous studies comparing plants that displayed stress-induced morphological differences after longer stress exposure. The small differences detected here could therefore be highly relevant for physiological differences between morphologically identical plants, and they would be suitable carriers of a long-term molecular memory that does not impact on plant development.

### Priming 'etches' H3K27me3 islands

**Figure 5:**
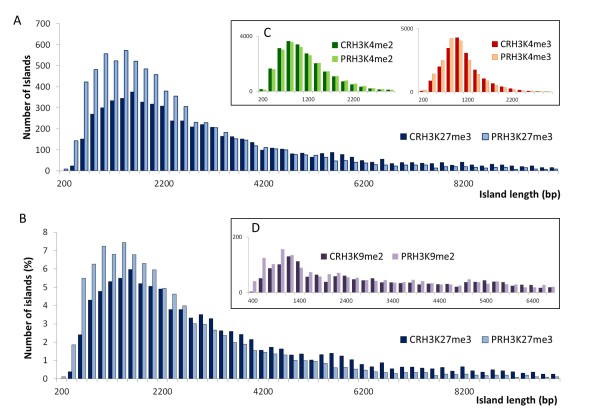
**Effect of priming on H3K27me3 island length distribution**. (**A**) Island length histograms plotting absolute number of H3K27m3 islands against island length in 200 bp length windows. (**B**) Island length histograms plotting percentage of H3K27m3 islands (relative to total island number in the sample) against island length in 200 bp windows. (**C**) Island length histograms for H3K4m2 and H3K4m3. (**D**) Island length histograms for H3K9m2. In all histograms values for non-primed root samples (CR) are given in dark colors, values for primed root samples (PR) are given in light colors.

Visual inspection of the histone modification profiles at high resolution revealed that differences in H3K4me2 and H3K4me3 most commonly consisted in higher peaks of existing islands in the primed samples. By contrast, the majority of differences in H3K27me3 occurred either at island edges or in 'valleys' within existing islands (see for example Figure 4). These sites already displayed low H3K27me3 occupancy in the non-primed samples and occupancy was further reduced in the primed samples. Priming-induced shortening and fractionation ('etching') of existing H3K27me3 islands explained why a higher number of H3K27me3 islands were found to cover less of the genome (compare Figure 3A, B). Indeed island length distribution profiles of primed and non-primed samples (Figure 5) showed that the increase of H3K27me3 island number in primed plants was only apparent in short islands (Figure 5A). Normalization to total island number revealed that the increase in the number of shorter H3K27me3 islands after priming was matched by a decrease in the number of long islands (Figure 5B). By contrast, island length distributions of the other modifications were similar between primed and non-primed plants (Figure 5C, D).

The observation of H3K27me3 island etching is interesting because to date little is known about the spatial characteristics of H3K27me3 removal. Establishment of H3K27me3 is thought to occur through a 'nucleation' and 'spreading' process, which involves recruitment of PCR2 complex members first to specific sequences and then to the neighboring regions by an autocatalytic process [37,38]. Valleys within H3K27me3 islands are then likely to reflect the merging of islands initiated at distinct nucleation sites. Here we found that a decrease of H3K27me3 in response to a mild salt treatment occurred primarily at island edges and in valleys within islands, thereby shortening existing islands and dividing longer islands into shorter ones. Thus removal of H3K27me3 appears to start from the youngest parts of the islands rather than the original nucleation regions thereby reversing the spreading of H3K27me3. Considering that the changes were observed within 24 h we can assume that they are due to active demethylation rather than low maintenance of the mark during replication. The first and so far only enzyme shown to function as a H3K27me3 demethylase in plants has recently been identified as REF6 [39] but kinetic and spatial properties of REF6-mediated demethyation remain to be studied. A functional genetics approach should now be used to test whether REF6 is required for the priming-induced changes in H3K27me3 profiles, and for the physiological priming effects.

### Transcription factors are preferred targets of priming-induced changes

To investigate which genes were targets of priming-induced changes we identified for all four modifications those islands and differential sites that mapped to genes resulting in gene lists for 'mapped islands' and 'mapped differences'. We then explored enrichment of functional annotations among genes carrying differential sites using the Database for Annotation, Visualization and Integrated Discovery (DAVID [40]). To compensate for any priming-independent functional bias among genes associated with certain histone modifications we scored enrichment of gene functions in 'mapped differences' not only over gene functions in the Arabidopsis genome (background 'Arabidopsis') but also over gene functions in the corresponding 'mapped islands' lists (backgrounds 'Islands Primed' and 'Islands Control'). The number of genes containing H3K9me2 islands was too small to extract statistically significant results but a highly significant enrichment of genes encoding transcription factors was found among genes that experienced priming-induced changes in H3K4 and H3K27. The approximately two-fold enrichment of transcription factors was represented by several functional terms extracted from different databases, and was independent of the chosen background or the length of upstream and downstream sequences included in the mapping. An example of gene numbers, enrichment and statistical parameters for the term 'transcriptional regulation' is shown in Table 2. The full dataset is provided as Additional file 2. Our finding that priming-induced differences in H3K27me3 were preferentially seen in transcription factors supports the notion of REF6 involvement because transcription factors were also found to be enriched among REF6-binding genes [39].

**Table 2:** Enrichment^a ^of genes functionally classified as 'transcriptional regulation'^b ^among genes that show priming-induced differences in H3K4me3 or H3K27me3

Modification	Sequence^c^	Background^d^	Count^e^	%^f^	Fold enrichment^g^	*P *value^h^
H3K4me3	Gene + 1,000 bp	Islands Primed	129	8.86	1.98	4.97E-14
		Islands Control	127	8.72	2	4.32E-14
		Arabidopsis	129	8.86	2.02	2.85E-14
	Gene + 100 bp	Islands Primed	127	8.78	1.97	1.15E-13
		Islands Control	123	8.51	1.96	3.63E-13
		Arabidopsis	129	8.92	2.04	1.73E-14

H3K27me3	Gene + 1,000 bp	Islands Primed	130	4.33	2.4	4.60E-21
		Islands Control	139	4.63	1.4	5.05E-06
		Arabidopsis	238	7.94	1.83	3.88E-21
	Gene + 100 bp	Islands Primed	124	4.65	1.42	4.25E-06
		Islands Control	76	2.85	1.43	4.74E-04
		Arabidopsis	258	9.68	2.25	7.03E-37

^a^As determined by DAVID [40]. For full dataset see Additional file 2.

^b^Term from SP PIR KEYWORDS.

^c^Length of up- and downstream sequences included in the mapping of islands and differences to genes.

^d^Background list used for identification of enriched functional terms in submitted gene list.

^e^Number of genes matching the annotation term in submitted gene list.

^f^Percentage of all genes in the submitted gene list.

^g^Relative enrichment compared to percentage in background.

^h^Statistical significance of enrichment.

### Priming-induced changes in H3K4me3 and H3K27me3 do not merely reflect simultaneous transcriptional responses to the priming treatment

**Figure 6:**
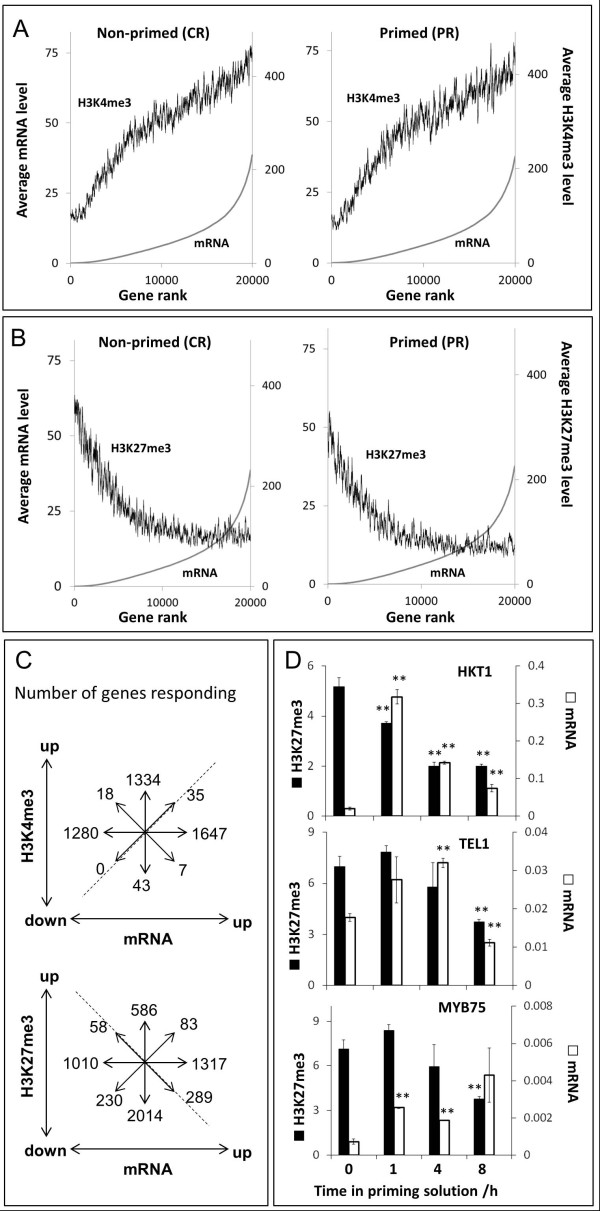
**Relationship between histone methylation and mRNA levels during and after priming**. (**A**, **B**) Genes on the x-axis were ranked according to mRNA levels determined by RNA-Seq. The mRNA profiles shown as smooth lines were generated from plotting for each gene on the x-axis the average mRNA values (right y-axis) over the neighboring genes with ranks of +/-100. Average values of histone modification levels (A: H3K4me3, B: H3K27me3) were plotted for the same genes (left y-axis). Relationships for non-primed root samples (CR) are shown in the graphs on the left, those for primed root samples (PR) are shown in the graphs on the right. (**C**) Numbers of genes that show an increase (up) or decrease (down) of mRNA level (x-axis) or histone modification level (y-axis) in response to the priming treatment (primed compared to non-primed roots). Note that the majority of changes observed immediately after the priming treatment do not show the expected positive (H3K4me3) or negative (H3K27me3) correlation between mRNA and histone modification (dashed lines). (**D**) Short-term kinetics of changes of mRNA and H3K27me3 levels in three genes (HKT1, TEL1, and MYB75) during the priming treatment. Relative enrichment of H3K27me3 (black bars) and mRNA levels (open bars) of selected genes in roots of *A. thaliana *seedlings were determined by qPCR over a time course of the first 8 h (x-axis) of the priming treatment (50 mM NaCl). H3K27me3 enrichment (left y-axis) was normalized to ChIP input and to a reference region in At5g56920. mRNA levels (right y-axis) were normalized to reference gene RpII. Bars show means ± SE of four pairwise ratios of two technical replicates of qPCR carried out with pooled root material from approximately 50 plants per time point. Significant differences to time point 0 are indicated with * for *P *<0.01.

Plants respond to acute salt/hyperosmotic stress with changes in the transcription of many genes. We were interested whether the observed changes in H3K4me3 and H3K27me3 simply mirrored simultaneous transcriptional responses to the priming treatment. We therefore sequenced mRNA isolated from the same plant roots used for the ChIP-Seq experiments (harvested immediately after the 24-h priming treatment). The mRNA sequence reads were mapped to the *A. thaliana *genome, and the mRNA levels obtained for each gene were plotted against cumulative read counts for H3K4me3 or H3K27me3 over the same genes, and ranked by mRNA level (Figure 6). Correlations were not apparent at single-gene level, but averaging over shifting windows of 200 genes reproduced the expected positive or negative correlation between gene expression and H3K4me3 or H3K27me3, respectively (Figure 6A, B). However, correlations were much weaker at high mRNA levels than at low mRNA levels, especially in the case of H3K27me3. Thus, mRNA levels of individual genes cannot be predicted from cumulative histone modification levels and *vice versa*. Furthermore, a lack of a dynamic relationship between gene activity and histone modification was apparent when the priming-induced changes in either parameter were compared for individual genes. As shown in Figure 6C the numbers of genes that showed changes in both mRNA level and histone modifications after priming was low and the number of genes that showed the expected correlation was even lower. Our results are in contrast to those of a reported a strong positive correlation between changes of mRNA and H3K4me3 in drought-stressed plants [25]. However, in that study H3K4me3 and mRNA levels were measured several days after the onset of dehydration stress and thus reflect a new steady-state representing phenotypic differences in growth and development of the plants.

The observed lack of correlation between changes in mRNA and changes in chromatin modifications could be due to a difference in the kinetics of transcriptional regulation and chromatin biochemistry. For example, many transcriptional responses to salt are fast and transient [41], while chromatin changes may be slower and more persistent. We therefore analyzed by qPCR mRNA and H3K27me3 levels of selected genes in roots of plants harvested during the first 8 h of the priming treatment. We found that changes in H3K27me3 were already detectable a few hours after salt addition. There was no consistent relationship between mRNA and H3K27me3 dynamics among the 10 genes analyzed (Additional file 1, Figure S4). However, for three genes (HKT1, TEL1, and MYB75) we found that rapid and transient induction at mRNA level was followed by a slower, long-lasting loss of H3K27me3 (Figure 6D).

To our knowledge, the kinetics of fast changes in histone modifications and mRNA immediately after a step change in the environment has not been explored before. Pulse-chase experiments in synchronized mammalian cells analyzed by mass spectrometry indicated that lysine tri-methylation is a relatively slow process requiring some 30 h to be re-established after DNA replication [42]. We found here that at least for some genes a decrease of H3K27me3 in response to a hyperosmotic stimulus already occurred within a few hours. This suggests that demethylation of H3K27me3 is a fast process that operates at a speed that is comparable to that of transcriptional regulation. Nevertheless the changes in specific H3K27me3 sites identified were in most cases not correlated with changes in mRNA, although in some genes they followed, and could have been triggered, by a fast transient change in transcription. Our findings underscore the fact that the exact relationship between histone modifications and transcription is still poorly understood. Both the reciprocal causality of chromatin modifications and transcriptional activity, and the temporal series of molecular events leading to changes in chromatin status are still under debate [43]. It will therefore be interesting to investigate in the future whether and how the changes in H3K27me3 observed here are mechanistically connected to other chromatin-based processes involved in dehydration-responsive gene transcription such as histone acetylation and/or nucleosome repositioning [44,45]. At this stage we conclude that a mild salt treatment causes rapid small changes in H3K4me3 and H3K27me3 that are superimposed onto the established steady-state correlation between transcript levels and histone modification status. The question then arises whether the priming-induced chromatin changes, once put into place, persist after removal of the original stimulus and whether they modulate gene transcription when the stimulus reoccurs.

### Priming-induced alterations of genome-wide H3K27me3 profiles are maintained during a 10-day growth period in control conditions

**Figure 7:**
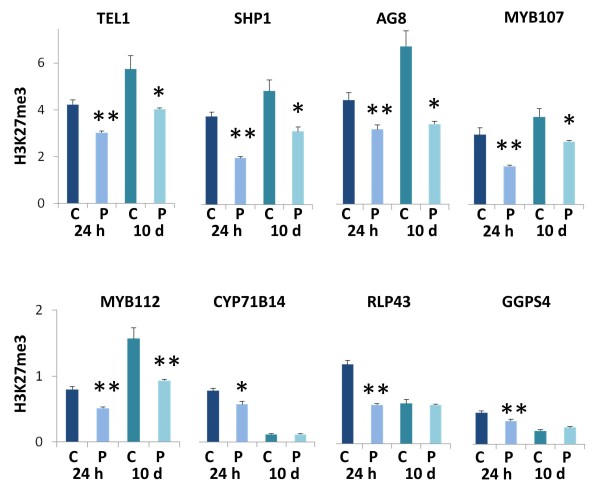
**Maintenance and loss of H3K27me3 marks 10 days after priming**. Average relative amounts of DNA amplified by qPCR from anti-H3K27me3 ChIP samples obtained from roots of primed (P, dark color) and non-primed (C, light colors) plants immediately after the 24-h priming treatment (24 h, blue) or 10 days later (10 d, turquoise). Each value was normalized to ChIP input and to constitutive reference region in At5g56920. Bars are means ± SE of three independently treated replicate plant batches each consisting of approximately 300 plants (same material as pooled for ChIP-sequencing). Significant differences between primed and non-primed plants are indicated with * for *P *<0.05 and ** for *P *<0.01.

**Figure 8:**
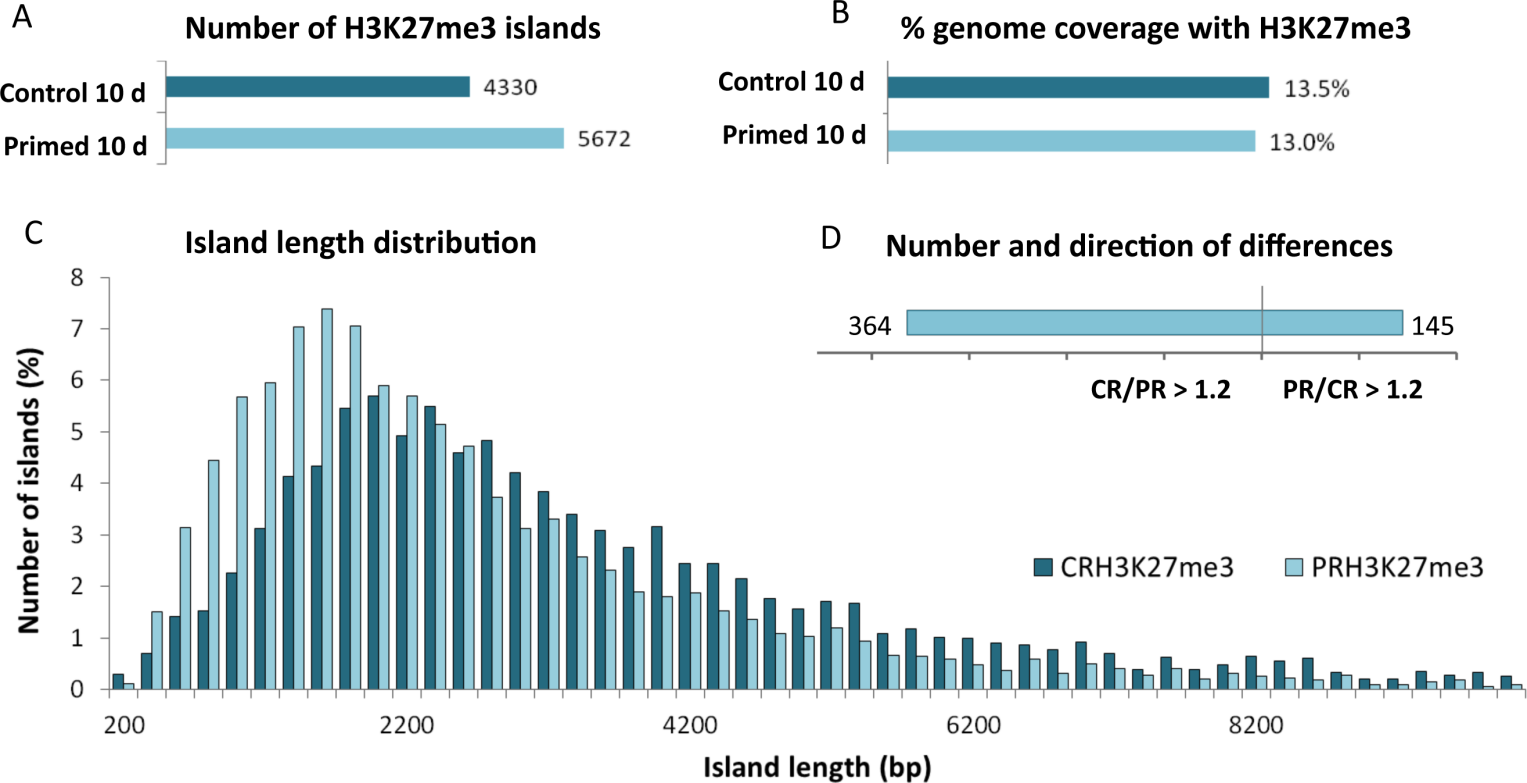
**Properties of genome-wide H3K27me3 profiles 10 days after priming**. Total number of islands (**A**), percentage genome coverage with islands (**B**), island length distribution (**C**) and number and direction of differences between primed and non-primed samples (**D**) of H3K27me3 in roots of non-primed (control, dark turquoise) and primed (light turquoise) plants after a growth period of 10 days in control conditions. Data were obtained from pooled root material representing three independently treated plant batches of approximately 300 plants each. Compare to H3K27me3 immediately after the priming treatment (Figure 3A-C and Figure 5B).

To investigate whether priming-induced changes in the chromatin were maintained over the extensive growth period following the priming treatment, primed and non-primed plants were transferred to hydroponics and allowed to grow in control conditions for 10 days. At this point chromatin was isolated from roots of three independently grown plant batches and subjected to ChIP and quality control as before. This analysis was limited to H3K27me3, the modification that was most strongly affected by the priming. ChIP-qPCR analysis of selected genes showed that for five out of nine genes a decrease in H3K27me3 measured immediately after the 24-h priming treatment was still apparent 10 days later (Figure 7). For those genes that showed no longer a difference between primed and non-primed samples after 10 days, very little DNA was recovered from both 10-day samples (primed and non-primed), suggesting that a priming-independent loss of H3K27me3 during plant maturation had cancelled the priming-induced mark. For HKT1 no DNA was recovered from the 10-day sample (see next section). Motivated by the qPCR results for individual genes, we pooled and sequenced the 10-day ChIP samples to identify long-term H3K27me3 changes at the genomic scale using the same methodology as before. All files obtained (aligned read counts, islands, and differences) are available as additional files for upload into IGB (Additional files 15, 16, 29, 30, and 37, see Methods). The total number of sequence reads from the 10-day samples was lower than from the 24-h samples and hence the landscapes had lost some of their depth. Nevertheless, the genome-wide profiles of the 10-day samples reproduced the basic features discovered in the 24-h samples (Figure 8). For example, H3K27me3 islands occurred in larger number while covering less of the genome in primed plants than in non-primed plants (Figure 8, AB). Accordingly, the length distribution of H3K27me3 islands in primed samples was still skewed towards more, smaller islands in the primed samples (Figure 8C), indicating that island fractionation was maintained during the growth period. Site-specific differences between primed and non-primed samples, identified by CHIPDIFF, although lower in total number, still showed a bias towards a decrease of H3K27me3 in the primed plants (Figure 8D).

**Figure 9:**
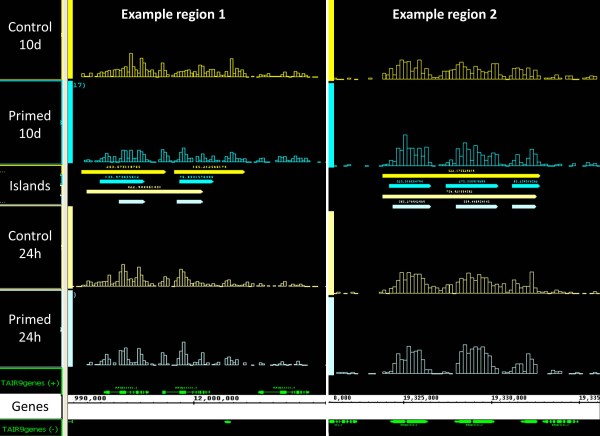
**Examples of H3K27me3 islands etching 24 h and 10 days after priming**. H3K27me3 profiles of primed and non-primed (control) root samples 24 h and 10 days after priming (screenshots of IGB display). Positions and lengths of islands identified by SICER are indicated with bars in the middle section. Note that in both example regions a long H3K27me3 island in the control samples is fractionated into a shorter island in the primed samples. This effect is still apparent after a 10-day growth period in control conditions.

The genome-wide high resolution profiles revealed many cases of island etching at the same position in 24-h and 10-day samples. Two examples are depicted in Figure 9. Comparison of positions of all differential sites between the 10-day and the 24-h samples resulted in a list of 102 genes with position-specific long-term changes in H3K27me3 (Table 3 and Additional file 1, Table S1). Interestingly, in many cases the length of the affected region was shorter after the 10-day growth period than immediately after the priming treatment. We conclude that the gaps in H3K27me3 islands that were generated by the priming treatment were transmitted through mitosis, but progressively 'filled' during growth in non-stressed conditions, probably due to PRC2-mediated spreading of H3K27me3 into the etched areas. Thus, priming-triggered demethylation of H3K27 at and within existing islands might require active maintenance in order to prevent fading of the molecular memory through H3K27me3 spreading. It would be interesting to investigate now whether repeated exposure to salt can prevent memory loss and eventually lead to the removal of entire H3K27me3 islands.

**Figure 10:**
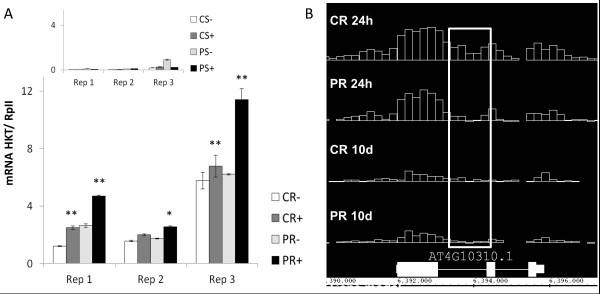
**Transcript and H3K27me3 profiles of HKT1 in primed and non-primed plants**. (**A**) mRNA levels of HKT1 (relative to constitutive gene RpII) determined by qPCR in roots of primed plants (PR, light grey and black bars) or non-primed plants (CR, white and dark grey bars) 10 days after priming and 4 h after application of 0 (-, control) or 80 mM NaCl (+, stress treatment). Inset shows very low expression of HKT1 in the shoots of the same plants. Results are shown separately for three independently primed and treated plant batches (Rep1-3) each consisting of pooled tissue from 12 plants. Bars are means ± SE of four pairwise ratios of two technical replicates. Significant differences between primed and non-primed plants for each condition (+/- salt) are indicated with * for *P *<0.05 or ** for *P *<0.01. (**B**) H3K27me3 profile over the HKT1 sequence in primed (PR) and non-primed (CR) root immediately after priming (24h) and 10 days later (10d) as displayed in IGB. The differential site identified by CHIPDIFF in the 24-h samples is marked with a white box.

**Figure 11:**
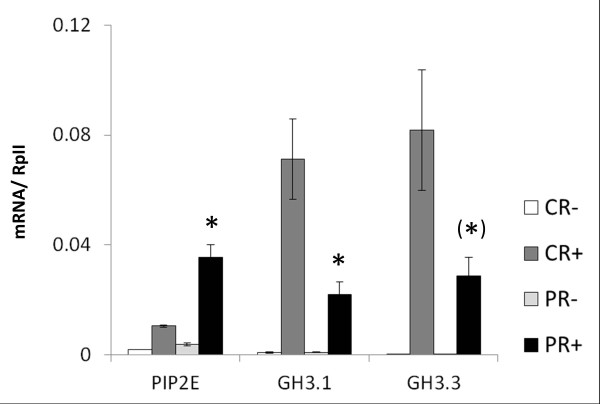
**Transcript profiles of PIP2E, GH3**.1, and GH3.3 in primed and non-primed plants. mRNA levels of PIP2E, GH3.1, and GH3.3 (relative to constitutive gene RpII) determined by qPCR in roots of primed plants (PR, light grey and black bars) or non-primed plants (CR, white and dark grey bars) 10 days after priming and 4 h after application of 0 (-) or 80 mM NaCl (+). Bars are means ± SE of three independently treated replicate plant batches each consisting of 12 plants. Significant differences between primed and non-primed plants for each condition (+/- salt) are indicated with * for *P *<0.05 and (*) for *P *= 0.06.

**Table 3:** Direction and length of priming-induced stable differences in H3K27me3

Gene ID	Description (from TAIR)	P/C^a^ 24 h^b ^	Length^c ^24 h/bp	Length 10 d/bp	P/C^a^ 10 d^d ^
AT1G05291	Unknown protein	Down	200	200	Down
AT1G09380	Integral membrane family/MtN21-related	Down	200	200	Down
AT1G12190	F-box family protein	Down	800	400	Down
AT1G12260	ANAC007; transcription factor	Down	600	400	Down
AT1G18710	AtMYB47 transcription factor	Down	600	200	Down
AT1G19800	TGD1; lipid transporter	Down	200	200	Down
AT1G30795	Hydroxyproline-rich glycoprotein family protein	Down	200	200	Down
AT1G31875	Unknown protein	Down	600	200	Down
AT1G47370	Toll-Interleukin-Resistance (TIR) domain	Down	600	200	Down
AT1G47786	Acyl-protein thioesterase-related	Down	400	200	Down
AT1G51460	ABC transporter family protein	Down	600	200	Down
AT1G51490	BGLU36 (beta glucosidase 36); hydrolase	Down	200	400	Down
AT1G52070	Jacalin lectin family protein	Down	200	200	Down
AT1G52140	Unknown protein	Down	1,800	200	Down
AT1G52410	TSA1; calcium ion binding/protein binding	Down	800	200	Down
AT1G56650	PAP1; transcription factor	Down	600	200	Down
AT1G57830	Toll-Interleukin-Resistance (TIR) domain	Down	400	400	Down
AT1G59722	Unknown protein	Down	1,000	200	Down
AT1G61630	ENT7 equilibrative nucleoside transporter	Down	800	600	Down
AT1G61750	Molecular_function unknown	Down	200	200	Down
AT1G62030	DC1 domain-containing protein	Up	200	400	Up
AT1G65342	Unknown protein	Down	200	200	Down
AT1G67710	ARR11; transcription factor/ two-component	Down	400	200	Down
AT1G69090	F-box family protein	Down	800	200	Down
AT1G69120	AP1 (Apetala 1); DNA binding	Down	800	200	Down
AT1G75920	Family II extracellular lipase 5 (EXL5)	Down	400	200	Down
AT2G14710	F-box family protein	Down	200	200	Down
AT2G14960	GH3.1	Up	400	200	Up
AT2G16220	F-box family protein	Down	600	200	Down
AT2G21890	CAD3 cinnamyl alcohol dehydrogenase	Down	400	400	Down
AT2G22180	Hydroxyproline-rich glycoprotein family protein	Down	400	200	Down
AT2G23170	GH3.3; indole-3-acetic acid amido synthetase	Up	400	200	Up
AT2G23171	Unknown protein	Down	200	200	Down
AT2G24205	ECA1 gametogenesis related family protein	Down	200	200	Down
AT2G25697	Unknown protein	Down	600	200	Down
AT2G25700	ASK3 (SKP1-like 3); ubiquitin-protein ligase	Down	600	200	Down
AT2G26580	YAB5 (YABBY5); transcription factor	Down	600	1,000	Down
AT2G30300	Nodulin-related	Down	200	200	Down
AT2G30760	Unknown protein	Down	200	200	Down
AT2G31083	CLE5 (Clavata3/ESR-related 5)	Down	1,200	200	Down
AT2G32870	Meprin and TRAF homology domain protein	Down	200	200	Down
AT2G34790	MEE23; FAD binding	Down	800	200	Down
AT2G39010	PIP2E; water channel	Down	600	400	Down
AT3G03200	Anac045; transcription factor	Down	400	200	Down
AT3G09390	MT2A; copper ion binding	Down	400	400	Down
AT3G16360	AHP4; histidine phosphotransfer kinase	Down	200	200	Down
AT3G18010	WOX1 (Wuschel related); transcription factor	Down	600	200	Down
AT3G18550	BRC1 (Branched 1); transcription factor	Down	1,800	200	Down
AT3G20160	Geranylgeranyl pyrophosphate synthase, putative	Up	600	200	Up
AT3G21840	ASK7 (SKP1-LIKE 7); ubiquitin-protein ligase	Down	400	400	Down
AT3G21850	ASK9 (SKP1-LIKE 9); ubiquitin-protein ligase	Down	400	200	Down
AT3G22080	Meprin and TRAF homology domain protein	Down	200	200	Down

AT3G25710	BHLH32; transcription factor	Down	600	200	Down
AT3G29260	Short-chain dehydrogenase/reductase family	Down	800	200	Down
AT3G29970	Germination protein-related	Down	800	400	Down
AT3G44780	Unknown protein	Down	1,000	200	Down
AT3G45560	Zinc finger (C3HC4-type RING finger) family	Down	600	200	Down
AT3G50480	HR4 (homolog of RPW8 4)	Down	600	200	Down
AT3G51200	Auxin-responsive family protein	Down	400	200	Down
AT3G55700	UDP-glucoronosyl/UDP-glucosyl transferase	Down	200	400	Down
AT4G00300	Fringe-related protein	Up	400	200	Up
AT4G01420	CBL5 (Calcineurin B-like protein 5)	Down	400	200	Down
AT4G01520	Anac067; transcription factor	Down	200	200	Down
AT4G04840	Methionine sulfoxide reductase domain protein	Down	1,000	200	Down
AT4G04890	PDF2; transcription factor	Down	800	200	Down
AT4G10220	Unknown protein	Down	1,000	200	Down
AT4G10350	ANAC070; transcription factor	Down	400	200	Down
AT4G11170	Disease resistance protein (TIR-NBS-LRR class)	Down	1,200	200	Down
AT4G12510	Lipid transfer protein (LTP) family protein	Down	600	400	Down
AT4G12520	Lipid transfer protein (LTP) family protein	Down	400	200	Down
AT4G13420	HAK5 (high affinity K+ transporter 5)	Up	400	200	Up
AT4G19730	Glycosyl hydrolase family 18 protein	Down	600	400	Down
AT4G19740	Catalytic/ cation binding/chitinase/ hydrolase	Down	1,600	200	Down
AT4G22030	F-box family protein	Down	200	200	Down
AT4G22070	WRKY31; transcription factor	Down	200	200	Down
AT4G28840	Unknown protein	Down	800	200	Down
AT4G29033	Encodes a defensin-like (DEFL) family protein.	Down	800	200	Down
AT4G30590	Plastocyanin-like domain-containing protein	Down	200	200	Down
AT5G04386	Unknown protein	Down	200	200	Down
AT5G11520	ASP3 (aspartate aminotransferase 3)	Down	400	400	Down
AT5G15160	bHLH family protein	Down	600	200	Down
AT5G15210	ATHB30; transcription factor	Up	200	400	Up
AT5G15800	SEP1 (Sepallata 1); transcription factor	Down	600	200	Down
AT5G17100	Molecular_function unknown	Down	400	400	Down
AT5G17810	WOX12 (Wuschel related); transcription factor	Down	1,600	600	Down
AT5G17960	DC1 domain-containing protein	Up	1,000	200	Down
AT5G24070	Peroxidase family protein	Down	200	200	Down
AT5G24820	Aspartyl protease family protein	Up	200	400	Up
AT5G24910	CYP714A1; electron carrier/ heme binding	Down	200	400	Down
AT5G25390	SHN2 (shine2); transcription factor	Down	1,000	200	Down
AT5G25990	Unknown protein	Down	200	200	Down
AT5G35770	SAP (sterile apetala); transcription factor	Down	1,000	400	Down
AT5G39560	Molecular_function unknown	Up	600	200	Up
AT5G40040	60S acidic ribosomal protein P2 (RPP2E)	Up	400	200	Up
AT5G40790	Unknown protein	Down	800	200	Down
AT5G42590	CYP71A16; electron carrier/ heme binding	Down	600	200	Down
AT5G43120	Tetratricopeptide repeat (TPR)-containing protein	Up	400	400	Up
AT5G45200	Disease resistance protein (TIR-NBS-LRR class)	Down	1,000	200	Down
AT5G46350	WRKY8; transcription factor	Down	400	400	Down
AT5G57785	Unknown protein	Down	800	400	Down
AT5G60010	FAD binding/calcium ion binding	Down	800	200	Down
AT5G60970	TCP5; transcription factor	Down	600	200	Down

^a^P/C: Direction of H3K27me3 change in primed versus non-primed plants.

^b^24 h: Immediately after the priming treatment.

^c^For exact genome coordinates see Additional File 1, Table S1.

^d^10 d: After 10 days of growth in control conditions.

### Priming alters the transcriptional response of the sodium transporter HKT1 to salt stress after a 10-day growth period in control conditions

The observation of a marked loss of H3K27me3 in HKT1 (At4g10310) during the priming treatment (Figure 6D) attracted our attention. HKT1 is a root-specific Na transporter that removes Na from the transpiration stream [46]. Indeed, lower shoot Na accumulation in primed plants after application of the second salt treatment (Figure 2B) mimicked the phenotype of *A. thaliana *mutant lines over-expressing HKT1 specifically in xylem parenchyma cells [47]. We therefore measured mRNA levels of HKT1 after application of the second salt treatment 10 days after the priming treatment. In accordance with previous findings, HKT1 displayed root specific expression in all plants. Importantly, in three independently primed plant batches HKT1 mRNA was always most abundant in the salt-treated primed plants (Figure 9). This was either due to a higher constitutive level (Figure 9A, replicate 1) or to stronger induction by salt in primed plants (Figure 9A, replicates 2 and 3). The experiment not only showed that the priming treatment still affected transcriptional responses after the 10-day growth period in control conditions, but also identified HKT1 as prime candidate for explaining at least one the physiological effects of the priming treatment. We were not able to recover enough DNA from anti-H3K27me3 ChIP from the individual replicate 10-day samples to allow detection of H3K27me3 in the differential HKT1 site by qPCR. However, the high-resolution profile obtained by ChIP-sequencing, while confirming very low H3K27me3 occupancy in the mature plants, still showed lower read counts in the primed sample than in the non-primed sample after 10 days (Figure 9B). The findings suggest that a persistent loss of H3K27me3 at HKT1 occurs in a very small number of cells that occupy a strategically important position in the root while HKT1 is silenced through other mechanisms in the rest of the plant. Indeed, HKT1 fulfills different functions depending on developmental stage and cell type [48,49], with shoot Na limitation being a consequence of HKT1 activity in root xylem parenchyma cells [47]. It is therefore conceivable that in adult plants H3K27me3 occupancy of HKT1 and changes thereof are also cell-type specific. Silencing of HKT1 in the shoots has been linked to distantly upstream tandem repeats and to siRNA-mediated DNA methylation within the HKT1 promoter [50,51]. Similar processes might silence HKT1 in most root cells of adult plants and preclude H3K27me3 occupancy. In this case very few cells would have contributed DNA to the anti-H3K27me3 ChIP sample thereby explaining low read count in the 10-day samples. Cell-type specific ChIP experiments are now needed to further consolidate the role of HKT1 in somatic salt stress memory.

### Several other genes also show altered transcriptional responses to the second salt treatment

Although over-expression of HKT1 alone could explain the low-sodium phenotype of primed plants, the 4-h salt treatment also represented an opportunity to investigate early transcriptional responses to a hyperosmotic stimulus. We therefore used the root mRNA samples from this treatment and time point to test for priming-dependent transcriptional responses of other genes. Twenty genes covering a range of functions were selected from Table 3 for qPCR analysis. Most of these genes had very low expression levels in both primed and non-primed plants independent of whether they were treated with salt or not (Additional file 1, Table S2). However, we found that PIP2E (At2g39010), encoding a plasma membrane aquaporin [52], was upregulated in the salt-treated plants and that this response was stronger in the primed than in the non-primed plants. Two other genes, GH3.1 (At2g14960) and GH3.3 (At2g23170), encoding auxin and jasmonate amino-acid conjugating enzymes respectively [53], were also upregulated by the salt treatment, but showed a weaker response in primed than in non-primed plants. Opposite effects of priming on the transcriptional responsiveness of PIP2E and HKT1 (more responsive) and on GH3.1 and GH3.3 (less responsive) were in accordance with opposite effects of priming on H3K27me3 in these genes, with PIP2E and HKT1 experiencing a decrease of H3K27me3 and GH3.1 and GH3.3 experiencing an increase. Whether this correlation reflects a direct causal relationship between H3K27me3 levels and transcriptional regulation during the second stress response needs now to be further investigated. Our results support the view that the priming-induced chromatin changes do not affect constitutive transcriptional activity but modulate access of stress-inducible transcriptional regulators thereby limiting any priming effects to reoccurring stress situations. The exact contributions of the identified differentially regulated genes to the physiological priming effect remain to be identified but they are likely to be related to water and hormone homeostasis during osmotic stress. Interestingly, GH3.1 is exclusively expressed in the root epidermis and hence in direct contact with the root environment [54].

In summary, we have identified four genes that show persistent priming-induced changes of H3K4me3 and altered transcriptional responses in response to a second salt treatment. Clearly this is just the beginning of a wider search for genes that could link the epigenetic memory to the physiological priming effects. This search needs to cover a range of stimuli and time points during the second stress exposure. In particular, it will be necessary to design a soil-free 'drought' experiment that produces clean root material to enable identification of those genes that underlie the main priming effect of enhanced drought tolerance (Figure 2C, D).

## Conclusions

A short treatment of *A. thaliana *seedlings with a moderate concentration of NaCl alters physiological responses of the adult plants to salt and drought stress, and causes small but significant changes in genome-wide profiles of four histone modifications. Priming-indued 'etching' of H3K27me3 islands is still apparent after a growth period of 10 days. The number of location-specific changes of H3K27me3 decreases during this period suggesting that the memory fades over time. Nevertheless persistent differences of H3K27me3 occur in >100 genes. One of these genes, encoding the Na-transporter HKT1, is more strongly induced in the primed plants than in the non-primed plants during a second salt treatment, which could explain the observed reduction of Na accumulation in salt-primed plants. The high-resolution genome-wide datasets generated in this study provide an essential resource for further exploration of the novel molecular features of somatic long-term memory in plants unveiled by our experiments.

## Materials and methods

### Plant growth and treatments

Batches of approximately 300 *Arabidopsis thaliana *(Col-0) seeds were germinated on vertical agar plates containing 70 mL of a minimally sufficient nutrient solution [55] supplemented with 3% sucrose (10 h light/14 h dark photoperiod, light intensity 120 μmol m^-2^s^-1^, 22°C). When seedlings had reached the four-leaf stage 5 mL of growth medium supplemented with 50 mM NaCl (or other concentrations as stated in the Results section) was applied directly to the roots (priming treatment). Control plants were treated in the same way using nutrient medium without NaCl added. Twenty-four hours later seedlings were transferred either to soil or to hydroponics. After 10 days of growth in control conditions, watering of soil-grown plants was stopped to induce drought stress. At the same time 80 mM NaCl was added to the hydroponics to induce salt stress. Plant tissues were harvested at several times during the protocol as indicated in the Results section.

### Measurement of tissue ion content

Ions were acid extracted from dried plant material by 24-h incubation with 2M HCl (1:100 w:v). The Na concentration in the extraction buffer was measured by spectrophotometry (410 flame photometer, Sherwood-Scientific Ltd., Cambridge, UK) and related to the dry weight of the plant material used.

### Extraction and immunoprecipitation of chromatin

Chromatin extraction and immunoprecipitation (ChIP) were carried out following published protocols [56]. In brief, tissue samples were incubated in 1% (w/v) formaldehyde for 15 min under vacuum. Cross-linking was stopped by adding 125 mM glycine, and tissues were rinsed, blotted dry, and frozen. Diluted chromatin extracts were incubated with antibodies against H3K4me2 (Diagenode pAb-035-050), H3K4me3 (Diagenode pAb-003-050), H3K9me2 (17-681, Millipore), or H3K27me3 (Diagenode, pAb-069-050). Immunoprecipitated chromatin-DNA (IP-DNA) or input chromatin-DNA was reverse cross-linked and residual protein was removed by proteinase K treatment. DNA was recovered by phenol/chloroform extraction and ethanol precipitation. As a quality control for successful ChIP, existence or absence of sequences previously found to be associated (positive control) or not (negative control) with certain histone modifications [32,34,57] in the ChIP samples was confirmed by semi-quantitative PCR. The primer pairs listed in Additional file 1, Table S3, recognized specific regions in the following genes: AT5G56920 (positive control) and AT5G56900 (negative control) for H3K27me3, AT1G24560 (positive and negative control) for H3K4me2/3, AT1G37110 (positive control) and AT2G05920 (negative control) for H3K9me2. Samples that had passed the quality control were used for further analyses.

### ChIP-qPCR and ChIP-Sequencing

Primers were designed to amplify specific regions of interest as stated in the Results section. Primer pairs are listed in Additional file 1, Table S3. ChIP DNA and input DNA samples were linearly amplified using GenomePlex Complete Whole Genome Amplification (WGA2, Sigma-Aldrich) following the manufacture instructions. Amplified samples were used as template for qPCR analysis carried out with Brilliant III SYBR Green qPCR kit (Stratagene) on a Mx3000 system (Stratgene). Ct values obtained were dually normalized to input and to reference region (same as 'positive controls' in quality control, see above). Sequencing of the ChIP DNA was carried out in the Glasgow Polyomics Facility (University of Glasgow). A DNA library was prepared using the ChIP-SEQ Sample Prep Kit (Illumina), according to the manufacturer's protocol, size selected on an agarose gel, amplified by PCR, and loaded onto separate lanes of GAIIx flow cells at a concentration of 12 pM. After cluster formation (Illumina Cluster station) the samples were sequenced (Illumina Genome Analyzer IIx) producing single 76 bp reads.

### mRNA extraction, RT-qPCR, and RNA-sequencing

RNA was extracted with RNeasy mini kit (Qiagen) according to the manufacture instructions and reverse transcribed to cDNA using QuantiTect® Reverse Transcription kit (Qiagen). qPCR analysis were carried out as described above. Several conventionally used reference genes were evaluated for stable expression across our experimental conditions using geNorm [58] and on the basis of this analysis RpII (At4g35800) was chosen as reference gene. Sequencing of the RNA was carried out in the Glasgow Polyomics Facility. RNA-seq libraries were prepared using mRNA 8-Sample Prep Kit (Illumina). Briefly, polyA RNA was isolated from 1 µg of total RNA, fragmented, and subjected to first- and second-strand cDNA synthesis with random primers. Subsequent sequencing procedures were the same as for DNA (see above).

### Analysis of ChIP-seq and RNA-seq data

*Read alignment*: For each sample, unaligned 76 bp reads in fastq format were generated with Casava version 1.7 (Illumina Inc.). Reads were aligned to the *A.thaliana *genome (TAIR9) using Bowtie version 0.12.5 [59] allowing for either unique or multiple read alignments with up to two mismatches in the first 54 bases. The alignment files in SAM/BAM format were then sorted, and duplicated reads with the same orientation removed using Samtools [59]. Alignment of sequence reads to unique targets in the *Arabidopsis thaliana *genome accommodated 76% to 94% of sequences for H3K4me2, H3K4me3, and H3K27me3, increasing to 79% to 97% when alignment to multiple targets was permitted. Only 42% of H3K9me2 reads could be aligned to unique targets, but due to preferential association of H3K9me2 with repeat sequences this number increased to over 74% when multiple alignments were included. Subsequent analyses were based on unique alignments for H3K4me2, H3K4me3, and H3K27me3, and on multiple alignments for H3K9me2. The final alignment positions of non-redundant reads were stored in BED files.

*Identification of histone modification domains*: Enriched histone modification domains ('islands') were calculated for each sample using SICER [35] version 1.03 with random background option (SICER-rb). A window size of 200 bp, an e-value of 0.1, and sample-specific DNA fragment sizes varying from 216 to 268 bp were used throughout the calculations. Based on a simulated alignment by Bowtie of 76-bp reads, synthetically generated by exhaustive partition of the entire genome, an effective genome fraction of 0.94 was used in all calculations. To determine the optimal internal gap length, SICER-rb was run with gaps of 0-5 window sizes (0-1,000 bp). For each histone modification the gap length producing the maximal aggregate score of all significant islands was then selected for further calculations. The optimal gap lengths were 0 for H3K4me2 and H3K4me3, 600 bp for H3K9me2, and 400 bp for H3K27me3. SICER scores for each 200-bp window were stored in WIG files (Additional files 3 to 16). The genomic coordinates of the obtained islands were stored in BED files (Additional files 17 to 30). Both file formats can be displayed in modern genome browsers.

*Identification of sites showing difference in histone modification levels*: To identify histone modification sites that differed between primed and non-primed samples we used CHIPDIFF software based on a hidden Markov model approach [36]. Throughout the calculations we used the same values for effective genome fraction, sample-specific fragment sizes and optimized window sizes as in the SICER-rb calculations (see above). Applying these parameters required a small change in the software code. For other parameters default values were used [36]. Separate lists of differences were generated using thresholds of 1.2, 1.5, and 2-fold changes. The genomic coordinates of the identified differences were stored in BED files. Files containing coordinates of >1.2-fold differences are provided as additional files for upload into genome browsers (Additional files 31 to 37). A BED file containing coordinates of *A. thaliana *genes (TAIR9 version) is also provided for upload into genome browser (Additional File 38).

*Mapping: *The identified islands and differences were mapped onto TAIR9 gene annotations using an in-house built script. A region was associated with a gene if its genomic coordinates overlapped with those of a gene including up- and downstream sequences of either 100 bp or 1,000 bp (separate lists). If two genes were assigned to an island/ the annotation of the closer one was selected. Lists of all mapped islands and differences were saved in Excel format for further analysis.

*Analysis of RNA-seq data*: From each sample unaligned single-end 76 bp reads in fastq format were generated with Casava version 1.7 (Illumina Inc.). Reads were aligned to the *A.thaliana *genome (TAIR9) using Tophat version 1.4.0 [60]. Differences in mRNA-levels between primed and non-primed samples were explored with Cuffdiff version 1.3.0 [61] using upper-quartile normalization, which resulted in a list of genes with mRNA levels (in FPKM), fold changes (log2 of FPKM-ratio), and statistical significance in form of false discovery rate. For subsequent analyses the list was filtered for FDR >0.05 and log2 (FPKM ratio) >1.

### Primary accession

ChIP-Seq and RNA-Seq raw data obtained in this study are available at ArrayExpress under series accession number E-MTAB-1663 and E-MTAB-1668, respectively.

## Abbreviations

AG8: AGAMOUS 8; AGI: Arabidopsis Genome Initiative; ATGOLS3: Arabidopsis Thaliana Galactinol Synthase 3; ATX1: Arabidopsis homolog of Trithorax 1; BAM: Binary Alignment/Map; BED: Browser Extensible Data; bp: Base pair;, ChIP: Chromatin Immuno-precipitation; ChIP-qPCR: ChIP followed by qPCR; ChIP-Seq: ChIP Chromatin followed by Sequencing; Col-0: Columbia-0; COR15A: Cold-regulated 15A; Ct: Cycle threshold; CR: Control roots; CYP71B4: Cytochrome P450 family71B4; DAVID: Database for Annotation Visualization and Integrated Discovery; DNA: Deoxyribonucleic acid; DW: Dry weight; FDR: False detection rate; FPKM: Number of RNA fragments per kilobase of gene model per million of aligned reads; FW: Fresh weight; GH3: Gretchen Hagen 3; GGPS4: Geranylgeranyl pyrophosphate synthase 4; H3K4: Lysine 4 in histone 3; H3K27: Lysine 27 in histone 3; H3K4me2: Histone H3 di-methylated at lysine 4; H3K4me3: Histone H3 tri-methylated at lysine 4; H3K27me3: Histone H3 tri-methylated at lysine 27; H3K9me2: Histone H3 di-methylated at lysine 9; HKT1: High-affinity K transporter 1; HMT: Histone methyl-transferase; IGB: Integrated genome browser; LRP1: Low response to phosphate 1; mRNA: messenger Ribo-Nucleic Acid; MYB: Myeloblastosis family; NaCl: Sodium chloride; nt: Nucleotide; qPCR: quantitative polymerase chain reaction; *P *value: Probability value; PIP2E: Plasma membrane intrinsic protein 2E; pM: pico-Molar; RNA-Seq; RNA isolation followed by sequencing; polyA: Polyadenylated; PR: Primed roots; PRC1: Polycomb repressive complex 1; REF6: Relative of early flowering 6; RpII: RNA polymerase II; RLP43: Receptor like protein 43; RT-PCR: Reverse-transcribed polymerase chain reaction; siRNA: Small interfering RNA; SAM: Sequence alignment/map; SE: Standard error; SHP1: Shatterproof 1; SOS5: Salt overly-sensitive 5; SP-PIR KEYWORDS: Swiss-Prot-Protein Information Resource Keywords; SUVH: Suppressor of variegation H; TAIR: The Arabidopsis Information Resources; TEL1: Terminal Ear1-Like 1; WGA: Whole genome amplification; WIG: Wiggle track format; w/v: Weight per volume.

## Competing interests

The authors declare that they have no competing interests.

## Authors' contributions

ES carried out all experimental work apart from RT-qPCR analysis of transcriptional responses to salt stress, which was carried out by GP. PH generated and analyzed the ChIP-Seq and RNA-Seq data. VC participated in project design, co-supervised the experimental work, and helped to draft the manuscript. AA designed and managed the project, and wrote the manuscript. All authors have read and approved the final manuscript.

## Description of additional files

The following additional data are available with the online version of this paper: Additional file 1 is a .pdf file containing Figures S1-4 and Tables S1-3. Figure S1 shows plants after long-term salt stress. Figure S2 presents the results of successful ChIP quality control. Figure S3 displays genome-wide histone modification landscapes. Figure S4 shows the kinetics of RNA and H3K27me3 levels for all genes tested. Table S1 details chromosome coordinates of priming-induced H3K27me3 differences recorded immediately after the 24-h priming treatment and 10 days later. Table S2 lists the genes that were tested for transcriptional changes after the second salt treatment. Table S3 provides sequences of all primers used in this study. Additional file 2 is a .xls file containing all results of functional enrichments as obtained by DAVID. Additional files 3, 4, 5, 6, 7, 8, 9, 10, 11, 12, 13, 14, 15, 16, 17, 18, 19, 20, 21, 22, 23, 24, 25, 26, 27, 28, 29, 30, 31, 32, 33, 34, 35, 36, 37 provide genome-wide histone modification profiles that can be uploaded into any modern genome browser (for example, IGB). Additional files 3, 4, 5, 6, 7, 8, 9, 10, 11, 12, 13, 14, 15, 16 are .wig files containing read counts over 200-bp windows for all histone modifications in all samples. Additional files 17, 18, 19, 20, 21, 22, 23, 24, 25, 26, 27, 28, 29, 30 are .bed files containing genome coordinates of identified islands for all histone modifications in all samples. Additional files 31, 32, 33, 34, 35, 36, 37 are .bed files containing genome co-ordinates of identified differences (primed/non-primed) for all histone modifications in all samples. The labeling of these files is self-explanatory (C: Non-primed; P: Primed; R: Roots; S: Shoots). All files are for 24-h samples, except those labeled 'Tendays'. Additional file 38 contains coordinates of *A. thaliana *Col-0 genes (TAIR9 version).

## Supplementary Material

Additional file 1 **Additional Figures and Table. Figure S1. Appearance of primed and non-primed plants after long-term salt stress**. Plants had been treated with either 0 (control, not primed, left), 50 mM (primed, center), or 100 mM NaCl (primed, right) for 24 h at seedling stage and subsequently grown for 10 days in hydroponics without salt. The solution was then supplemented with 80 mM or not (control, no salt) and plants photographed 10 days later. No difference in salt tolerance was apparent between primed and non-primed plants. **Figure S2. Example of a successful ChIP quality control**. Based on published histone methylation profiles primers were designed to amplify regions that are enriched (positive controls) or devoid (negative controls) of H3K4me2, H3K4me3, H3K9me2, or H3K4me3. (Note that no region was found that was exclusively associated with H3K4me2.) ChIP samples from roots (R) or shoots (S) of primed (50) or non-primed (C) plants obtained with antibody against H3K9me2 (A), H3K27me3 (A), H3K4me2 (M2), or H3K4me3 (M3) were used as template as well as ChIP input DNA (I) and ChIP without antibodies (NA). For primer pairs see Table S3. **Figure S3. Genome-wide histone modification landscapes in primed and non-primed plants**. Genome-wide profiles of read counts for H3K4me2 (green), H3K4me3 (red), H3K9me2 (purple), and H3K27me3 (blue) in roots samples of primed (PR) and non-primed (CR) plants displayed in the Integrated Genome Browser (IGB). **Figure S4. Kinetics of H3K27me3 and mRNA after salt application**. Relative enrichment of H3K27me3 (black bars, left y-axis) and mRNA levels (open bars, right y-axis) of nine genes in roots of *A. thaliana *seedlings were determined by qPCR over a time course of 8 h (x-axis) after application of 50 mM NaCl (priming treatment). H3K27me3 levels (left y-axis) were normalized to ChIP input and to reference region in At5g56920. mRNA levels (right y-axis) were normalized to reference gene RpII. For details see main text and Figure 6D. **Table S1. Chromosome coordinates of priming-induced H3K27me3 differences. Table S2. Expression levels of selected genes in root RNA 4 h after second salt treatment. Table S3. Sequences of primers used in this study** Click here for file

Additional file 2 **Enrichment of functional terms among genes with mapped differences in H3K4me3 and H3K27me3 as determined by DAVID**. Enrichment calculated over genes with mapped islands in control (background C) or primed samples (background P). Upstream and downstreams sequences included were either 100 bp or 100 pb. Click here for file

Additional file 3 **Reads200_CRH3K4me2.wig**. WIG file containing sequence read counts over 200 bp windows in sample 24h CRH3K4me2. Click here for file

Additional file 4 **Reads200_CRH3K4me3.wig**. WIG file containing sequence read counts over 200 bp windows in sample 24h CRH3K4me3 Click here for file

Additional file 5 **Reads200_CRH3K9me2.wig**. WIG file containing sequence read counts over 200 bp windows in sample 24h CRH3K9me2 Click here for file

Additional file 6 **Reads200_CRH3K27me3.wig**. WIG file containing sequence read counts over 200 bp windows in sample 24h CRH3K27me3 Click here for file

Additional file 7 **Reads200_CSH3K4me2.wig**. WIG file containing sequence read counts over 200 bp windows in sample 24h CSH3K4me2 Click here for file

Additional file 8 **Reads200_CSH3K4me3.wig**. WIG file containing sequence read counts over 200 bp windows in sample 24h CSH3K4me3 Click here for file

Additional file 9 **Reads200_PRH3K4me2.wig**. WIG file containing sequence read counts over 200 bp windows in sample 24h PRH3K4me2 Click here for file

Additional file 10 **Reads200_PRH3K4me3.wig**. WIG file containing sequence read counts over 200 bp windows in sample 24h PRH3K4me3 Click here for file

Additional file 11 **Reads200_PRH3K9me2.wig**. WIG file containing sequence read counts over 200 bp windows in sample 24h PRH3K9me2 Click here for file

Additional file 12 **Reads200_PRH3K27me3.wig**. WIG file containing sequence read counts over 200 bp windows in sample 24h PRH3K27me3 Click here for file

Additional file 13 **Reads200_PSH3K4me2.wig**. WIG file containing sequence read counts over 200 bp windows in sample 24h PSH3K4me2 Click here for file

Additional file 14 **Reads200_PSH3K4me3.wig**. WIG file containing sequence read counts over 200 bp windows in sample 24h PSH3K4me3 Click here for file

Additional file 15 **Reads200_TendaysCRH3K27me3.wig**. WIG file containing sequence read counts over 200 bp windows in sample 24h CRH3K27me3 Click here for file

Additional file 16 **Reads200_TendaysPRH3K27me3.wig**. WIG file containing sequence read counts over 200 bp windows in sample 10d PRH3K27me3 Click here for file

Additional file 17 **Islands_CRH3K4me2-G600.bed**. BED file containing coordinates of islands as determined by SICER software in sample 24h CRH3K4me2. (G: optimized gap size in bp.) Click here for file

Additional file 18 **Islands_CRH3K4me3-G600.bed**. BED file containing coordinates of islands as determined by SICER software in sample 24h CRH3K4me3. (G: optimized gap size in bp.) Click here for file

Additional file 19 **Islands_CRH3K9me2-G400.bed**. BED file containing coordinates of islands as determined by SICER software in sample 24h CRH3K9me2. (G: optimized gap size in bp.) Click here for file

Additional file 20 **Islands_CRH3K27me3-G600.bed**. BED file containing coordinates of islands as determined by SICER software in sample 24h CRH3K27me3. (G: optimized gap size in bp.) Click here for file

Additional file 21 **Islands_CSH3K4me2-G600.bed**. BED file containing coordinates of islands as determined by SICER software in sample 24h CSH3K4me2. (G: optimized gap size in bp.) Click here for file

Additional file 22 **Islands_CSH3K4me3-G600.bed**. BED file containing coordinates of islands as determined by SICER software in sample 24h CSH3K4me2. (G: optimized gap size in bp.) Click here for file

Additional file 23 **Islands_PRH3K4me2-G600.bed**. BED file containing coordinates of islands as determined by SICER software in sample 24h PRH3K4me2. (G: optimized gap size in bp.) Click here for file

Additional file 24 **Islands_PRH3K4me3-G600.bed**. BED file containing coordinates of islands as determined by SICER software in sample 24h PRH3K4me3. (G: optimized gap size in bp.) Click here for file

Additional file 25 **Islands_PRH3K9me2-G400.bed**. BED file containing coordinates of islands as determined by SICER software in sample 24h PRH3K9me2. (G: optimized gap size in bp.) Click here for file

Additional file 26 **Islands_PRH3K27me3-G600.bed**. BED file containing coordinates of islands as determined by SICER software in sample 24h PRH3K27me3. (G: optimized gap size in bp.) Click here for file

Additional file 27 **Islands_PSH3K4me2-G600.bed**. BED file containing coordinates of islands as determined by SICER software in sample 24h PSH3K4me2. (G: optimized gap size in bp.) Click here for file

Additional file 28 **Islands_PSH3K4me3-G600.bed**. BED file containing coordinates of islands as determined by SICER software in sample 24h PSH3K4me3. (G: optimized gap size in bp.) Click here for file

Additional file 29 **slands_TendaysCRH3K27me3-G400.bed**. BED file containing coordinates of islands as determined by SICER software in sample 10d CRH3K27me3. (G: optimized gap size in bp.) Click here for file

Additional file 30 **Islands_TendaysPRH3K27me3-G400.bed**. BED file containing coordinates of islands as determined by SICER software in sample 10d PRH3K27me3. (G: optimized gap size in bp.) Click here for file

Additional file 31 **Differences_PvsC_RH3K4me2.bed**. BED file containing coordinates for differences determined by CHIPDIFF software between primed (P) and non-primed (C) 24h RH3K4me2 samples. Click here for file

Additional file 32 **Differences_PvsC_RH3K4me3.bed**. BED file containing coordinates for differences determined by CHIPDIFF software between primed (P) and non-primed (C) 24h RH3K4me3 samples. Click here for file

Additional file 33 **Differences_PvsC_RH3K9me2.bed**. BED file containing coordinates for differences determined by CHIPDIFF software between primed (P) and non-primed (C) 24h RH3K9me2 samples. Click here for file

Additional file 34 **Differences_PvsC_RH3K27me3.bed**. BED file containing coordinates for differences determined by CHIPDIFF software between primed (P) and non-primed (C) 24h RH3K27me3 samples. Click here for file

Additional file 35 **Differences_PvsC_SH3K4me2.bed**. BED file containing coordinates for differences determined by CHIPDIFF software between primed (P) and non-primed (C) 24h SH3K4me2 samples. Click here for file

Additional file 36 **Differences_PvsC_SH3K4me3.bed**. BED file containing coordinates for differences determined by CHIPDIFF software between primed (P) and non-primed (C) 24h SH3K4me3 samples. Click here for file

Additional file 37 **Differences_PvsC_TendaysRH3K27me3.bed**. BED file containing coordinates for differences determined by CHIPDIFF software between primed (P) and non-primed (C) 10d RH3K27me3 samples. Click here for file

Additional file 38 **TAIR9genes.bed**. BED file containing coordinates of *A. thaliana *genes. Click here for file
