# Supplementary material for: Hyperosmotic priming of Arabidopsis seedlings establishes a long-term somatic memory accompanied by specific changes of the epigenome
Source: Genome Biol. 2013 Jun 14;14(6):R59. doi: 10.1186/gb-2013-14-6-r59 (PMC3707022; doi:10.1186/gb-2013-14-6-r59)
Supplement: Additional file 1 — Additional Figures and Table. Figure S1. Appearance of primed and non-primed plants after long-term salt stress. Plants had been treated with either 0 (control, not primed, left), 50 mM (primed, center), or 100 mM NaCl (primed, right) for 24 h at seedling stage and subsequently grown for 10 days in hydroponics without salt. The solution was then supplemented with 80 mM or not (control, no salt) and plants photographed 10 days later. No difference in salt tolerance was apparent between primed and non-primed plants. Figure S2. Example of a successful ChIP quality control. Based on published histone methylation profiles primers were designed to amplify regions that are enriched (positive controls) or devoid (negative controls) of H3K4me2, H3K4me3, H3K9me2, or H3K4me3. (Note that no region was found that was exclusively associated with H3K4me2.) ChIP samples from roots (R) or shoots (S) of primed (50) or non-primed (C) plants obtained with antibody against H3K9me2 (A), H3K27me3 (A), H3K4me2 (M2), or H3K4me3 (M3) were used as template as well as ChIP input DNA (I) and ChIP without antibodies (NA). For primer pairs see Table S3. Figure S3. Genome-wide histone modification landscapes in primed and non-primed plants. Genome-wide profiles of read counts for H3K4me2 (green), H3K4me3 (red), H3K9me2 (purple), and H3K27me3 (blue) in roots samples of primed (PR) and non-primed (CR) plants displayed in the Integrated Genome Browser (IGB). Figure S4. Kinetics of H3K27me3 and mRNA after salt application. Relative enrichment of H3K27me3 (black bars, left y-axis) and mRNA levels (open bars, right y-axis) of nine genes in roots of A. thaliana seedlings were determined by qPCR over a time course of 8 h (x-axis) after application of 50 mM NaCl (priming treatment). H3K27me3 levels (left y-axis) were normalized to ChIP input and to reference region in At5g56920. mRNA levels (right y-axis) were normalized to reference gene RpII. For details see main text and Figure 6D. Table S1. Chromosome [file gb-2013-14-6-r59-S1.DOCX]

**
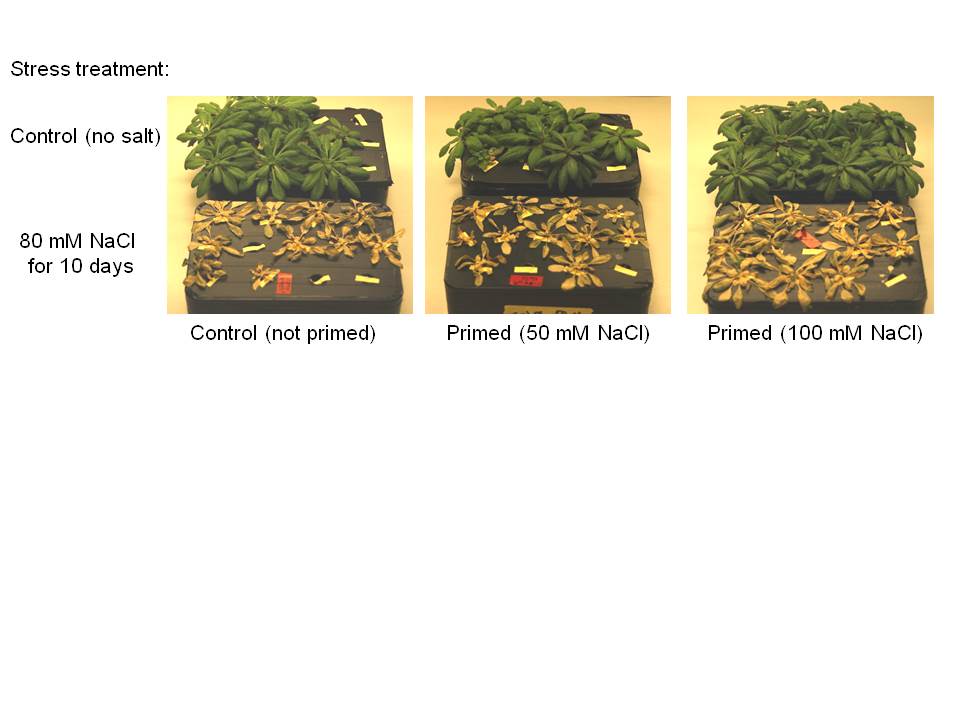
**

**Figure S1: Appearance of primed and non-primed plants after long-term salt stress**

Plants had been treated with either 0 (control, not primed, left), 50 mM (primed, centre) or 100 mM NaCl (primed, right) for 24 hours at seedling stage and subsequently grown for ten days in hydroponics without salt. The solution was then supplemented with 80 mM or not (control, no salt) and plants photographed 10 days later. No difference in salt tolerance was apparent between primed and non-primed plants.

.


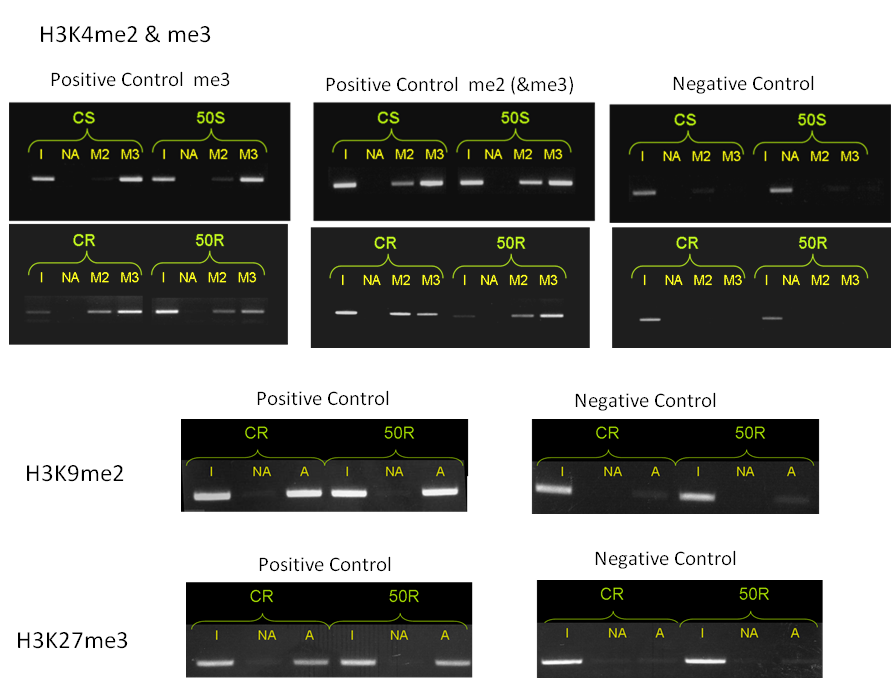


**Figure S2: Example of a successful ChIP quality control.**

Based on published histone methylation profiles primers were designed to amplify regions that are enriched (positive controls) or devoid (negative controls) of H3K4me2, H3K4me3, H3K9me2 or H3K4me3. [Note that no region was found that was exclusively associated with H3K4me2.] ChIP samples from roots (R) or shoots (S) of primed (50) or non-primed (C) plants obtained with antibody against H3K9me2 (A), H3K27me3 (A), H3K4me2 (M2) or H3K4me3 (M3) were used as template as well as ChIP input DNA (I) and ChIP without antibodies (NA). For primer pairs see Table S3.

**
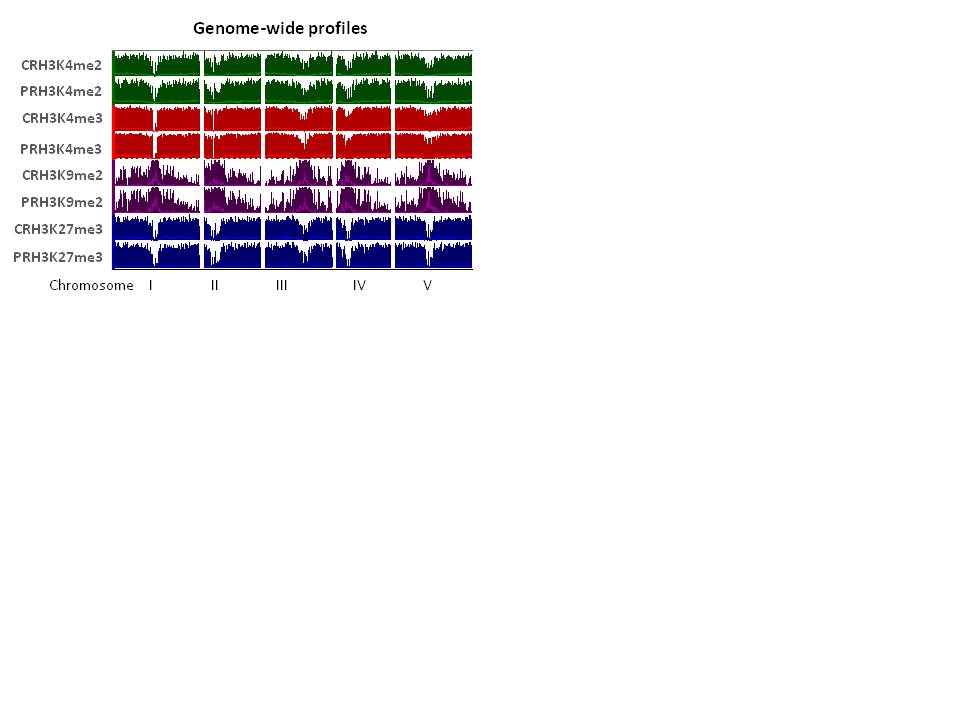
**

**Figure S3: Genome-wide histone modification landscapes in primed and non-primed plants**

Genome-wide profiles of read counts for H3K4me2 (green), H3K4me3 (red), H3K9me2 (purple) and H3K27me3 (blue) in roots samples of primed (PR) and non-primed (CR) plants displayed in the Integrated Genome Browser (IGB).


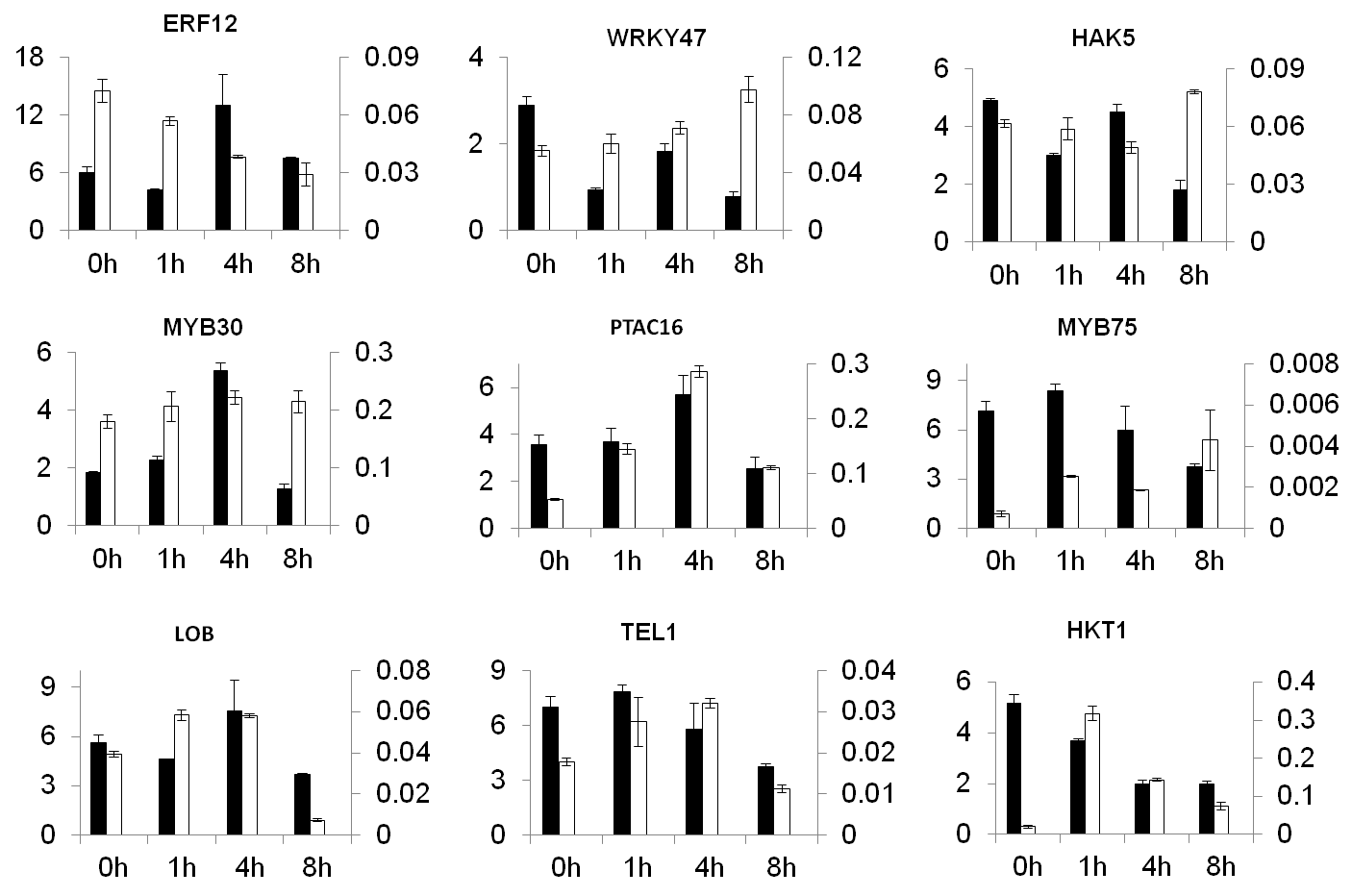


**Figure S4: Kinetics of H3K27me3 and mRNA after salt application**

Relative enrichment of H3K27me3 (black bars, left y-axis) and mRNA levels (open bars, right y-axis) of nine genes in roots of *A. thaliana* seedlings were determined by qPCR over a time course of eight hours (x-axis) after application of 50 mM NaCl (priming treatment). H3K27me3 levels (left y-axis) were normalized to ChIP input and to reference region in At5g56920. mRNA levels (right y-axis) were normalized to reference gene RpII. For details see main text and Figure 6D.

**Table S1: Chromosome coordinates of priming-induced H3K27me3 differences**


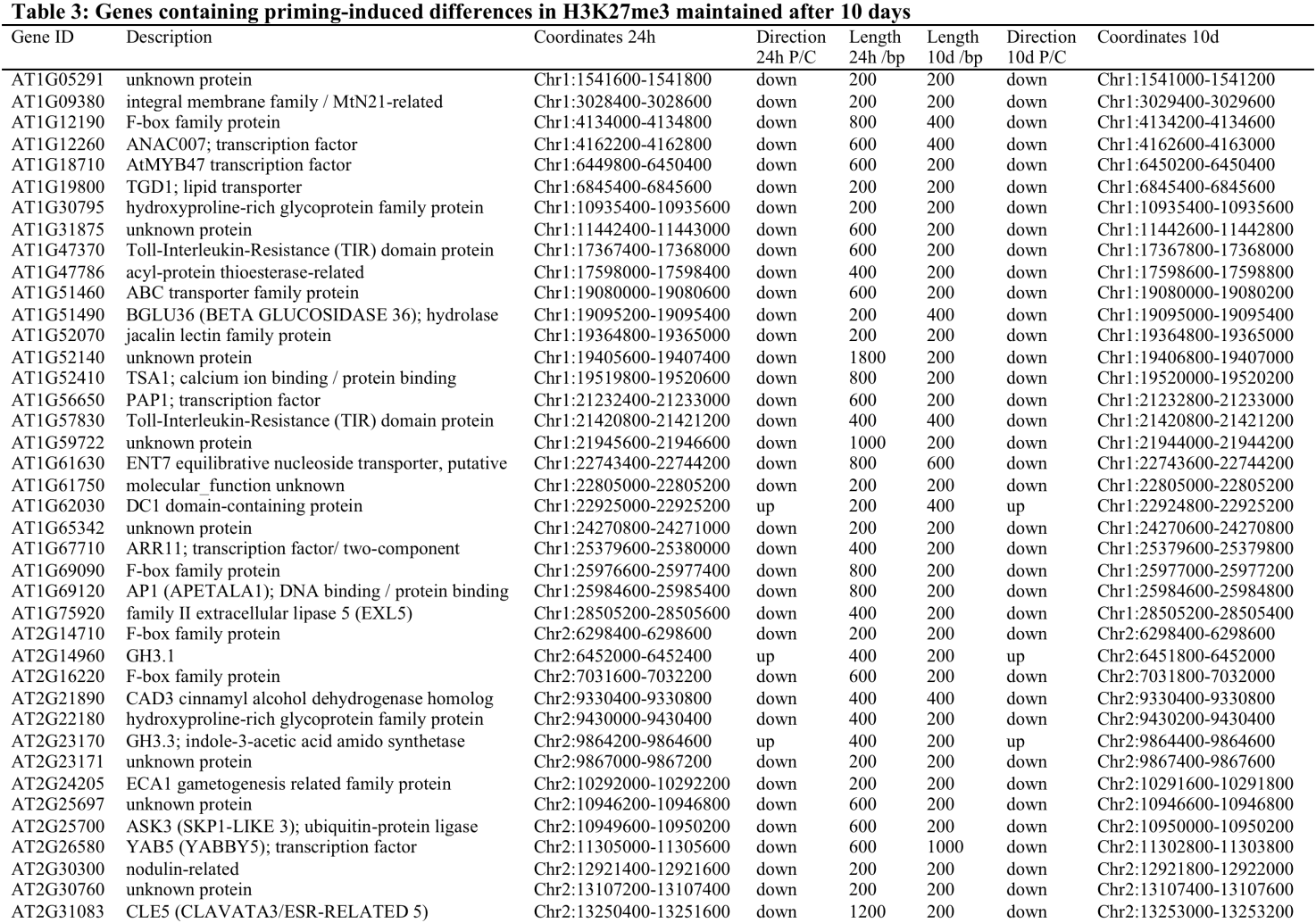

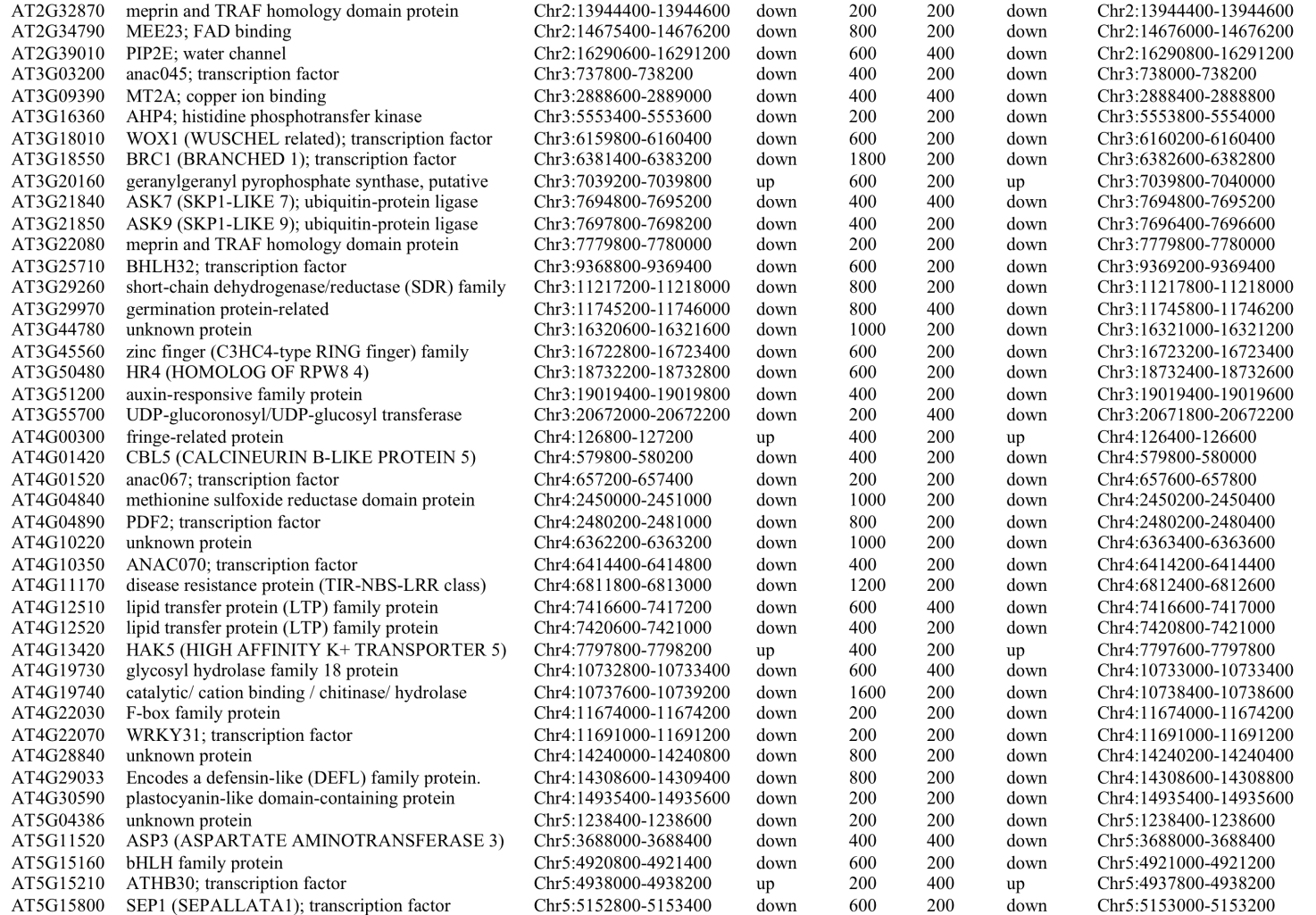


**Table S1 cont.**


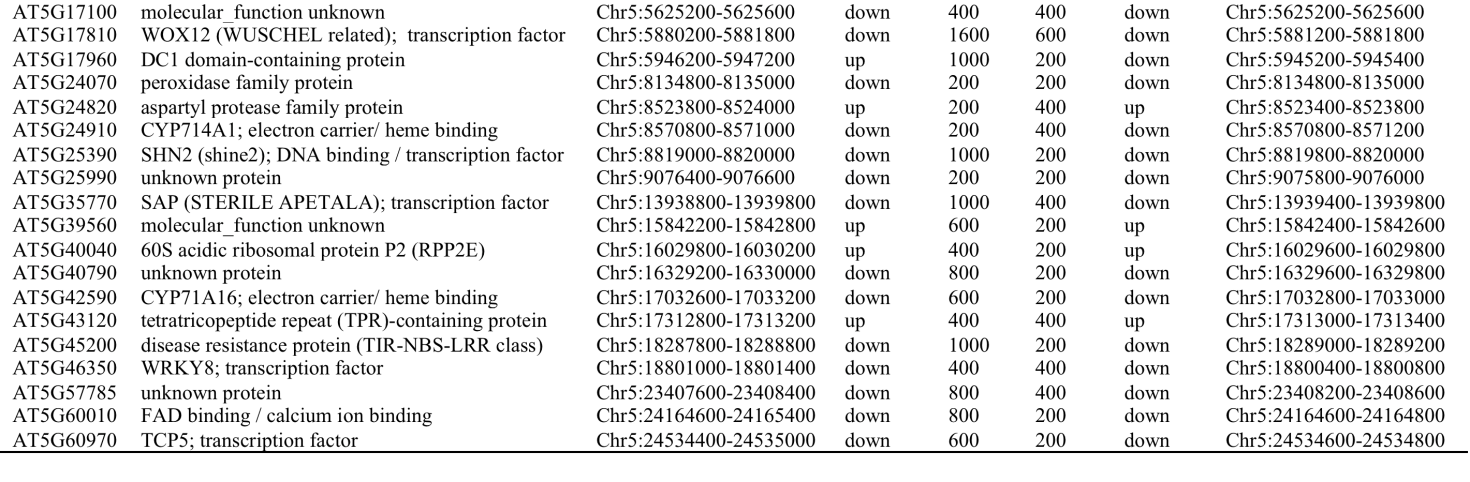


**Table S2: Expression levels of selected genes in root RNA 4 hours after 2^nd^ salt treatment**

**Table S3: Sequences of primers used in this study (F: Forward, R: Reverse)**

| Primer sequence | Primer name | Gene AGI | Usage |
| --- | --- | --- | --- |
| ATAGCTAGCTGATGGGTCAAGTC | At4g35800 -F | AT4G35800 | Reference gene RNA |
| GTTTTGAGTGTTTTGAAAAGGATT | At4g35800 -R | “ | “ |
| GGAGCTAATAGCGGAGCTTG | ChipCont1-1F | AT1G24560 | Positive Control H3K4me3 |
| TCCTTCAATGCTTCATCACG | ChipCont1-1R | “ | “ |
| GCACAGAGTTTGGTGGAAGAG | ChipCont1-2F | AT1G24560 | Positive Control H3K4me2 |
| CACCATCCCTCAAACCATTC | ChipCont1-2R | “ | “ |
| ACCAAGTCTAGCATGGGAGAGA | ChipCont1-3F | AT1G24560 | Negative Control me2-3 |
| TCGAGGGTTTGATTTACATGG | ChipCont1-3R | “ | “ |
| CATCATCCGCGTCAATCAC | H3K9me2 3-1F | AT2G05920 | Negative Control H3K9me2 |
| GAAACTCAGGAGTACGCG | H3K9me2 3-1R | “ | “ |
| AAGAGAGCTGGCAGAAGCAGTTGA | H3K9me2 1-1F | AT1G37110 | Positive Control K9me2 |
| ACGCCCTTTACCTTGACCTCCTTT | H3K9me2 1-1R | “ | “ |
| TGTGTGGAAGGGTCTTGTGGACTT | H3K9me2 2-2F | AT4G03745. | Positive Control K9me2 |
| AACTTACATGTTTGCGGGCACGAG | H3K9me2 2-2R | “ | “ |
| GGAATCAGAAATTCCAACAGG | H3K27me3-1-1F | AT5G56900 | Negative Control H3K27me3 |
| TTGATGCTCTTCGTGCACTT | H3K27me3-1-1R | “ | “ |
| AAGCAATGCCGTATTGATCC | H3K27me3-1-2F | AT5G56920. | Positive Control H3K27me3 |
| GCTGCACTTCAACAGCTTCA | H3K27me3-1-2R | “ | “ |
| GAGAAGCTAGCAATACGCAACG | MYB75-F | AT1G56650 | 24h H3K27me3 |
| TGCTATTAAAGGTTGTGCATGA | MYB75-R | “ | “ |
| **Table S3 cont.** |  |  |  |
| GAATTGTCTACTTATATTGTAC | CYP71B14 -F | AT5G25180 | 24h/10d H3K27me3 |
| CCATCATAAATTGTCAACGA | CYP71B14 -R | “ | “ |
| ACTGCTGGCCACCTGGTACT | RLP43-F | AT3G28890 | 24h/10d H3K27me3 |
| CTTCATCAATGGGGAAGCTG | RLP43-R | “ | “ |
| GATGAGTTGTCACTAGGGATCC | SHP1 -F | AT3G58780 | 24h/10d H3K27me3 |
| CAATGTTAGACAAAGTCATCCG | SHP1 -R | “ | “ |
| CGATCTTCTAAGCTAGATTTGA | AG8-F | AT4G18960 | 24h/10d H3K27me3 |
| CAAATATTCCCACTAATGTTAGTG | AG8-R | “ | “ |
| TGTGAATCATTTCAACTGCACA | GGPS4 F | AT2G18640 | 24h/10d H3K27me3 |
| GAGCCACTCAACATCCACAA | GGPS4R | “ | “ |
| GAGGGAGAGAGTTCTCGGAATG | MYB112F | AT1G48000 | 24h/10d H3K27me3 |
| CTATAGCATGTGCTTAAGGAG | MYB112R | “ | “ |
| GTGAGATTATCATCAAGAGC | MYB107F | AT3G02940 | 24h/10d H3K27me3 |
| GCCTAGAACCTATCTAATCAAACA | MYB107R | “ | “ |
| CCATTTCTTCAGTGGCTAATG | SOS5 H3K4me3-F | AT3G46550 | ChIP-Seq H3K4me3 /me2 |
| TCCAGTCATTACCGGCGGAG | SOS5 H3K4me3-R | “ | “ |
| CGCCTGATTTCCACAGTCTTG | LRP1 H3K4me3-F | AT5G12330 | ChIP-Seq H3K4me3 /me2 |
| CCACGAAACGCAGTATCTTCAC | LRP1 H3K4me3-R | “ | “ |
| CCGACCCGTTTCAAGTTACC | SCARECROW-LIKE H3K4me3-F | AT5G66770 | ChIP-Seq H3K4me3 /me2 |
| TCCACCGCCGAAATCAGATA | SCARECROW-LIKE H3K4me3-R | “ | “ |
| GAGAGACCCAAGAGTCCAAGAC | MYB30-F | AT3G28910 | Time-course ChIP |
| CTACTTTAGGTGGTAATGAGTC | MYB30-R | “ | “ |
| AGGCTTCACGAGGAGAATCA | WRKY47-F | AT4G01720 | Time-course ChIP |
| AGCTAATCTCGGTGCCACAT | WRKY47-R | “ | “ |
| GCAGGATATGCGACACATGA | HAK5-F | AT4G13420 | Time-course ChIP |
| TCGGAGGTGTTTTCCTCTGC | HAK5-R | “ | “ |
| CACGGAAACTTGAAATATTTCAT | MYB75-F | AT1G56650 | Time-course ChIP |
| GACGTTGATCAACTTTGGAGTC | MYB75-R | “ | “ |
| CGGTTCTTGATCGTGTTCATT | LOB-F | AT2G42440 | Time-course ChIP |
| ACTCTGAATTGGATCAGCACTTG | LOB-R | “ | “ |
| GCTGAGATACGAGATCCTTGG | ERF12-F | AT1G28360 | Time-course ChIP |
| GGTGGTTGAGGTCGAGAGAG | ERF12-R | “ | “ |
| GTTTCGACCTCAGACGCATT | PTAC16-F | AT3G46780 | Time-course ChIP |
| AGAGATAGTCAATGGCTGAGA | PTAC16-R | “ | “ |
| GGAAGATCCGAGTTGCAGAG | TEL1-F | AT3G26120 | Time-course ChIP |
| TATAGCACACGAATCCATGTG | TEL1-R | “ | “ |
| CACTCCCATGGATTGAGGAA | HKT1-K27me3-F | AT4G10310 | Time-course ChIP |
| TTGTGGAAAGTTTGACAAGCA | HKT1-K27me3-R | “ | “ |
| CCAATACTGGGCTGCTTAGA | RNA-MYB30-F | AT3G28910 | Time-course RNA |
| GATGAGAATCTTGATTGACT | RNA-MYB30-R | “ | “ |
| CAACATGAGGATGTACCTCA | RNA-WRKY47-F | AT4G01720 | Time-course RNA |
| GCTCTAACGGAAACCCTAGC | RNA-WRKY47-R | “ | “ |
| CTCCGGGATATAGACTTGTCCCT | HAK5-F | AT4G13420 | Time-course RNA |
| GCAATGTTTGCTGATCTAGGTCA | HAK5-R | “ | “ |
| GTCCAAGGCATGGAGGATTAACG | MYB75-F | AT1G56650 | Time-course RNA |
| GGTCGGACCGCAAATGACGTC | MYB75-R | “ | “ |
| CTGAGATACGAGATCCTTGGA | ERF12-F | AT1G28360 | Time-course RNA |
| CGCAGCGAACCAGAGCTGTTG | ERF12-R | “ | “ |
| GGAGTCACAGTGGACGGTTT | PTAC16-F | AT3G46780 | Time-course RNA |
| CTTCGGACGTTCTTCTTTCG | PTAC16-R | “ | “ |
| ACCATGGAAGATCCGAGTTG | TEL1-F | AT3G26120 | Time-course RNA |
| ATCCATGGGGAGATACACGA | TEL1-R | “ | “ |
| TTGGTTGGATCGTTGTTTCA | HKT1-F | AT4G10310 | Time-course RNA |
| CTTTCGGTGATTGAAATGAG | HKT1-R | “ | “ |
| TTTTCCGTGTTCATGGTTCA | PIP2E-F | AT2G39010 | Salt stress RNA |
| TCATTGCACCAGCTCTCAAC | PIP2E-R | “ | “ |
| CTTCGAGTTCATCCCTCTCG | GH3.1-F | AT2G14960 | Salt stress RNA |
| TGACTCGTCGGTCTTGTCAG | GH3.1-R | “ | “ |
| TCGGATAAAACCGATGAAGC | GH3.3-F | AT2G23170 | Salt stress RNA |
| TCAACGACTCCTCCATTTCC | GH3.3-R | “ | “ |
